# Genome-Wide Transcriptome Analysis of Cotton (*Gossypium hirsutum* L.) Identifies Candidate Gene Signatures in Response to Aflatoxin Producing Fungus *Aspergillus flavus*

**DOI:** 10.1371/journal.pone.0138025

**Published:** 2015-09-14

**Authors:** Renesh Bedre, Kanniah Rajasekaran, Venkata Ramanarao Mangu, Luis Eduardo Sanchez Timm, Deepak Bhatnagar, Niranjan Baisakh

**Affiliations:** 1 School of Plant, Environmental and Soil Sciences, Louisiana State University Agricultural Center, Baton Rouge, LA, 70803, United States of America; 2 Southern Regional Research Center, USDA-ARS, New Orleans, LA, 70124, United States of America; University of Nebraska-Lincoln, UNITED STATES

## Abstract

Aflatoxins are toxic and potent carcinogenic metabolites produced from the fungi *Aspergillus flavus* and *A*. *parasiticus*. Aflatoxins can contaminate cottonseed under conducive preharvest and postharvest conditions. United States federal regulations restrict the use of aflatoxin contaminated cottonseed at >20 ppb for animal feed. Several strategies have been proposed for controlling aflatoxin contamination, and much success has been achieved by the application of an atoxigenic strain of *A*. *flavus* in cotton, peanut and maize fields. Development of cultivars resistant to aflatoxin through overexpression of resistance associated genes and/or knocking down aflatoxin biosynthesis of *A*. *flavus* will be an effective strategy for controlling aflatoxin contamination in cotton. In this study, genome-wide transcriptome profiling was performed to identify differentially expressed genes in response to infection with both toxigenic and atoxigenic strains of *A*. *flavus* on cotton (*Gossypium hirsutum* L.) pericarp and seed. The genes involved in antifungal response, oxidative burst, transcription factors, defense signaling pathways and stress response were highly differentially expressed in pericarp and seed tissues in response to *A*. *flavus* infection. The cell-wall modifying genes and genes involved in the production of antimicrobial substances were more active in pericarp as compared to seed. The genes involved in auxin and cytokinin signaling were also induced. Most of the genes involved in defense response in cotton were highly induced in pericarp than in seed. The global gene expression analysis in response to fungal invasion in cotton will serve as a source for identifying biomarkers for breeding, potential candidate genes for transgenic manipulation, and will help in understanding complex plant-fungal interaction for future downstream research.

## Introduction

Aflatoxins represent the group of four polyketide-derived mycotoxins (B1, B2, G1 and G2) that are highly toxic and carcinogenic chemicals produced as secondary metabolites from toxigenic isolates of the saprophytic fungi *Aspergillus flavus* and *A*. *parasiticus* [1–6]. Aflatoxin B1 is the most widely occurring structure that is carcinogenic to humans and animals [2–4]. Aflatoxins cause suppression of the immune system, cancer, retardation in growth, and in extreme cases death of both humans and animals. Aflatoxins have the ability to contaminate variety of crops such as corn, cotton, peanut and tree nuts during their growth and development, accounting to an estimated economic loss of ~$270 M annually worldwide [4], [5], [7]. The occurrence of aflatoxin in agricultural products is highly prohibited. U.S. Food and Drug Administration (FDA) has imposed strict regulations on the levels of aflatoxin contamination in foods and feeds; the permitted aflatoxin levels in human food and milk is 20 parts per billion (ppb) and 0.5 ppb, respectively [8], but for the cereals, nuts and dried fruits, aflatoxin standards are more stringent, which is 4 ppb for total aflatoxin content and 2 ppb for aflatoxin B1 [8], [9]. The cottonseeds alone contribute ~15% of the income of the farmers from cotton. The contamination of cottonseed with aflatoxin is of high concern to the cotton industry because cottonseeds are used as a preferred meal to dairy cows due to their high protein content, and cottonseeds are also used for oil production. Further, cows fed with contaminated cottonseeds can metabolize the aflatoxin B1 to M1 (hydroxylated derivative of metabolized aflatoxin B1), which in their milk will ultimately affect humans [8]. The prices of cottonseeds are largely determined by the content of aflatoxin present. Aflatoxin contamination is a major problem in the arid cotton growing regions of Arizona, the Imperial Valley of California, South Texas, and to extent in Louisiana in the U.S., and accounts to high annual economic losses.

Considering the declining economy of the cottonseed industry due to the infection of cotton by *A*. *flavus*, it is highly important to take necessary steps to manage aflatoxin contamination in cotton. Both pre- and post-harvest strategies have been used to lessen the aflatoxin contamination in cotton and other crops. Pre-harvest strategies include control of insect pests and proper irrigation to manage aflatoxin contamination. The post-harvest strategies include control of storage conditions that are less favorable to fungal growth, and detoxification of aflatoxin from contaminated seeds and grains [10]. Some plant metabolites, such as linoleic acid derivative 13(S)-hydroperoxide, are known to inhibit aflatoxin synthesis [8], [11]. Further, the bio-competition by application of atoxigenic strains *A*. *flavus* and/or *A*. *parasiticus* to outcompete toxigenic strains in the fields has been shown to be an effective strategy to reduce the aflatoxin contamination [5], [8], [12]. Atoxigenic strains of *Aspergillus* were reported to reduce the contamination of aflatoxin by ~70–90% in cotton and peanut [13–15]. This bio-competition strategy is of utmost importance in cotton because cotton has limited genetic diversity, and to date, no aflatoxin-resistant genotype is available in cotton [4], [8].

The defense responses in plants depend on the type of pathogen [6], [16]. Among different mechanisms, defense responses in plants are known to be regulated by the phytohormones, such as salicylic acid (SA), jasmonic acid (JA), ethylene (ET), cytokinin (CK) and auxins [6], [16], [17]. As a general rule, plant resistance to biotrophic pathogens is controlled by SA. In contrast, the resistance to necrotrophic pathogens is controlled by JA- and ET-dependent signaling pathways [6], [16], [17]. Moreover, resistance to necrotrophic fungal pathogens is known to be quantitative in nature and regulated by multiple genes [6], [18]. Toxigenic strain of *A*. *flavus* is characterized with the features of a necrotrophic fungal pathogen [6]. It is essential to develop germplasm that can resist the fungal invasion and/or shut down toxin production for long-term control of *A*. *flavus* infections [4], [19]. However, conventional breeding for resistance to *A*. *flavus* in cotton has been handicapped due to the unavailability of the genetic resistance in the available cotton gene pool. Genetic engineering of cotton with genes induced or upregulated in response to *A*. *flavus* infection will provide a promising approach to develop cotton varieties resistant to *A*. *flavus*. This necessitates detail investigation into the host-pathogen interaction to identify genes that are induced in cotton in response to *A*. *flavus* invasion or by toxin production. Further, understanding the largely unknown molecular basis of bio-competition strategy in controlling toxigenic *A*. *flavus* infection using atoxigenic strain of *A*. *flavus* could lead to identification of candidate genes for their use in manipulation of *A*. *flavus* resistance in cotton. Strategies such as small-scale expressed sequence tag (EST) library sequencing and oligonucleotide microarray have been used for the identification of genes induced or regulated in response to *A*. *flavus* infection in crops, such as maize, peanuts and cotton [4], [20], [21]. These small-scale targeted strategies based on the identification one or a few genes are not sufficient to understand the complex host-pathogen interaction responses [22]. Therefore, to identify key regulators in the interaction of *A*. *flavus* infection with cotton, it is necessary to discover genes on a global scale using high-coverage transcriptome analysis approach. We report here the identification of differentially expressed/regulated genes in the pericarp and seed tissues of cotton in response to *A*. *flavus* infection with an objective to understand the complex genetics involved in defense response of cotton to both toxigenic and atoxigenic strains of *A*. *flavus* infection.

## Materials and Methods

### 1. Fungal Culture Preparation and Cotton Boll Inoculation

Fungal cultures of toxigenic (AF13) and atoxigenic (AF36) strains of *A*. *flavus* were prepared as described earlier [4]. Briefly, the strains were grown on maltose extract agar medium at 30°C for a week. Conidia were harvested by scrapping the mycelium in nine ml of potato dextrose broth, and the suspension was adjusted to a concentration of 10^4^ conidia/ml.

Cotton variety ‘Coker 312’ was grown in the greenhouse for the present study as described earlier [4]. A hole to a depth of 5–10 mm was made in the center of one of the locules (L1) of cotton bolls (28–30 dpa) with the help of a 3 mm dia cork borer. Ten L of the conidia suspension was applied into the hole with the help of a Pasteur pipet. Bolls inoculated with only potato dextrose broth (PDB) without conidia served as the control. Pericarp and fiber-free seeds from non-inoculated and inoculated locule (L1) and adjacent/distal (Adj) locules of cotton bolls were harvested in liquid nitrogen at 6, 24, 48, and 72 h after inoculation, and stored at -80°C for RNA isolation. Three bolls each from two different plants (biological replicates) were used for each treatment.

### 2. RNA Extraction, Library Preparation and Sequencing

The total RNA was separately extracted from seed and pericarp tissues from L1 and Adj locules collected at each time point by using Spectrum total RNA isolation kit (Sigma-Aldrich, St. Louis, MO). RNA quantity and integrity were assessed as described earlier [4].

For library preparation, 2 μg of RNA from each different time points and replications were mixed for each tissue and experimental condition in order to minimize the cost of library preparation. Altogether, six libraries–non-inoculated pericarp (NIP) and seed (NIS), Pericarp (NTP) and seed (NTS) inoculated with atoxigenic strain, and pericarp (TP) and seed (TS) inoculated with toxigenic strain were prepared as per the manual of Illumina RNA-library construction kit. The libraries were single-end sequenced using the Illumina HiSeq-2000 platform at the sequencing facilities of the Iowa State University, Johnston, IA.

### 3. Read Filtering and Sequence Assembly

The single-end raw short Illumina sequencing reads (100 bp) were subjected to filtering to obtain high quality reads for downstream analysis. The raw reads were filtered and trimmed for adapter contamination and low quality, ambiguous and uncalled nucleotide bases. The reads containing more than 5% of uncalled bases and of average quality < = 20 over a window size of 5 bp in 5’ to 3’ direction were discarded. The filtering and trimming of the raw reads were performed by an in-house pipeline developed with Perl and Python programming. Subsequently, the high quality reads were assembled de novo using Trinity (release 2013-02-25) [23] with parameters of k-mer size 25, minimum contig length 200 bp and min_kmer_cov 2. The assembly was performed individually for reads of each of the six libraries. The overlapping k-mers were assembled into linear transcripts followed by clusters of overlapping transcripts. The transcripts for alternative spliced form and paralogous genes from these overlapping transcripts were obtained. All bioinformatics data analysis was performed using the Louisiana State University High Performance Computing resource SuperMike-II configured with 16 CPUs. After the assembly was performed for each library, exactly duplicate (100% similar) transcripts were removed to determine the total unigenes. We have used the term “transcript” here to describe individual sequence assembly and “unigene” to denote the longest transcript from a particular alternatively spliced isoforms cluster.

### 4. Functional Annotation

The assembled transcripts were subjected to functional annotation using homology search against publicly available databases, such as *G*. *raimondii* protein database (http://phytozome.jgi.doe.gov/pz), *G*. *arboreum* protein database (http://cgp.genomics.org.cn/page/species/index.jsp), NCBI's non-redundant (nr) plant nucleotide sequence database (http://www.ncbi.nlm.nih.gov), and UniProtKB (SwissProt and Tr-EMBL plant sequences) database (http://www.uniprot.org). The homology search was performed using BLASTx algorithm [24] against the databases at an E-value cut-off of 1e-05. If the annotation from different databases conflicted with each other, priority was given to the match with *G*. *raimondii* protein database, NR and UniprotKB, in that order. The Kyoto Encyclopedia of Genes and Genomes (KEGG; http://www.genome.jp/kegg) database was utilized for assigning biological pathways to the transcripts. Further, based on the sequence similarity with *G*. *raimondii* protein database, GO annotations for biological process, molecular function and cellular component were assigned to the assembled transcripts. The GO enrichment analysis was performed with agriGO analysis toolkit [25] with default P-value and false discovery rate (FDR). For identification of enrichment of metabolic pathways, the PathExpress analysis tool with criteria of P< 0.05 was used [26].

### 5. Mapping Reads to Reference Sequence

The cleaned reads from each library were mapped individually to the D genome *G*. *raimondii* (http://www.phytozome.net/) and the A genome *G*. *arboreum* (http://cgp.genomics.org.cn/page/species/index.jsp) [27], [28] using Tophat (version 2.0.9) spliced aligner [29] and Bowtie2 aligner [30] with number of threads set to 10. The unaligned reads were mapped by Bowtie2 that split these into smaller segments for realigning and finding potential spiced sites. Mapped and unmapped reads were reported as BAM files, which were used for downstream analysis.

### 6. Differential Gene Expression Analysis

Differentially expressed genes from the six RNA-Seq libraries were identified by using respective BAM files for alignment with Tophat-Cufflink pipeline (version 2.1.1) that produced the transcript assembly [31], [32]. The read counts were normalized as Fragments per Kilobase of Transcripts per Million mapped fragments (FPKM). The assembly files created across infected and uninfected control conditions were pooled in a single file using Cuffmerge for differential expression analysis. Finally, the pooled file from Cuffmerge was fetched to Cuffdiff for calculating expression level and statistical significance of genes across control and inoculated conditions. Cuffdiff employed a blind dispersion model, which conservatively treated all conditions as a replicate of each other in the absence of transcripts from biological replicates as is the case in the present study [31], [32]. The codes used in Cuffdiff for identifying DEGs under NIP vs TP were as follows: cuffdiff-o cuffdiff_out_NIP_TP-b GraimondiiGenome.fa-p 10-L NIP,TP—max-bundle-frags 10000000—FDR-u merged.gtf accepted_hits_NIP.bam accepted_hits_TP.bam (Generalized code: cuffdiff-o cuffdiff_out_NIP_TP-b genome.fa-p 10-L NIP,TP-u merged.gtf accepted_hits_NIP.bam accepted_hits_TP.bam). The same code was used for other experimental conditions. The heatmaps for gene expression analysis (log2 fold change) were plotted using heatmap.2 function within R package (version 3.1.2). Three-way comparisons–NI vs NT, NI vs T, and NT vs T were performed to understand the modulation of gene expression between different experimental conditions for both pericarp and seed. The (digital) expression of 10 selected genes showing maximum log2 fold change under *A*. *flavus* infection in comparison to non-inoculated control was validated by reverse-transcription PCR using gene specific primers (**S1 Table; sheet 1**) following the method described earlier [4].

## Results and Discussion

### 1. Illumina Sequencing, Quality Control and Alignment to the Reference Genome

Sequencing of the six libraries generated 911,040,814 reads of 100 bp long resulting in a total of 91.1 Gbp sequence data (**Table 1)**. The average quality of the reads in the libraries after filtering was >33. There was negligible amount of adapter/primer contamination in the reads. Altogether, 907,357,848 high quality reads were obtained from all six libraries, which totaled to 90.73 Gbp sequencing data (**Table 1)**. The raw sequences have been deposited in the NCBI SRA database (http://www.ncbi.nlm.nih.gov/sra; Biopoject accession PRJNA275482). Out of high quality filtered reads, 59.6% mapped to the D genome of cotton (*Gossypium raimondii*), whereas 75.6% mapped to the A genome (*G*. *arboreum*) across all six libraries. The higher percentage of alignment of the reads to the A genome could be due to its higher genome size as compared to the D genome.

**Table 1:** Sequence and assembly statistics of six cotton libraries with/without *A*. *flavus* infection.

Tissue/library	Pericarp			Seed			
Parameter	NIP*	NTP	TP	NIS	NTS	TS	Total
# Raw single-end reads	163,090,907	138,340,968	196,958,025	193,242,949	137,348,154	82,059,811	911,040,814
Bases sequenced (Gbp)	16.30	13.83	19.69	19.32	13.73	8.205	91.1
Sequence coverage	65.24X	55.34X	78.78X	77.3X	54.94X	32.82X	364.42X
# Cleaned reads used in assembly	162,591,684	137,657,801	196,080,070	192,530,847	136,780,468	81,716,978	907,357,848
# Assembled unique transcripts	99,772	107,294	122,657	100,510	82,896	73,440	586,569
# unigenes	31,425	35,831	40,010	31,635	28,019	23,638	190,558
Transcript N50 (bp)	1887	1842	1976	1927	1703	1787	1864
Transcript N90 (bp)	701	647	699	705	634	658	675
Maximum transcript size (bp)	15441	14102	15414	15383	10154	14933	15441
Minimum transcript size (bp)	201	201	201	201	201	201	201
Average transcript Size (bp)	1339.58	1275.85	1351.4	1353.22	1217.53	1278.19	1307.8
Transcriptome size (Mbp)	133.65	136.89	165.75	136.01	100.92	93.87	767.11

*NIP = non-inoculated pericarp

NTP = atoxigenic pericarp; TP = toxigenic pericarp; NIS = non-inoculated seed; NTS = atoxigenic seed; TS = toxigenic seed.

### 2. De Novo Sequence Assembly

**Fig 1:**
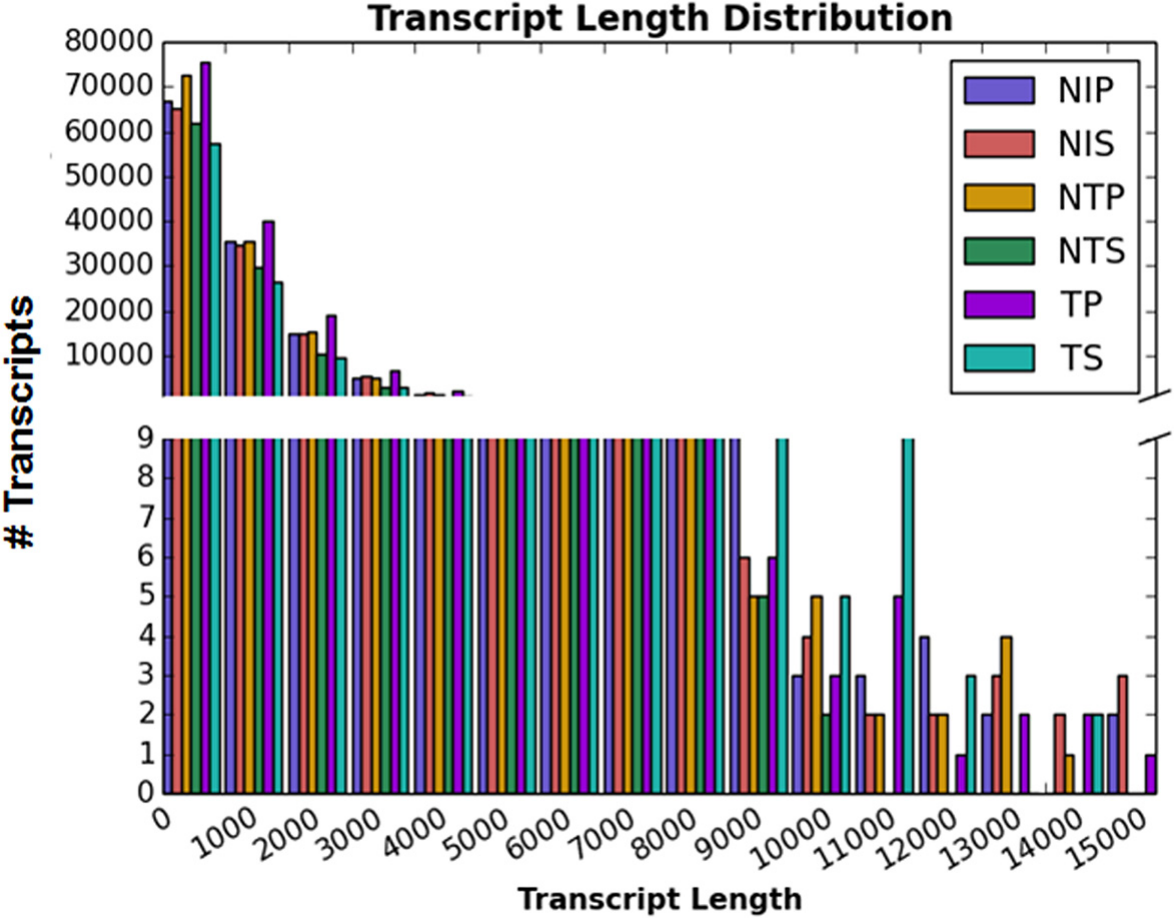
Lengthwise (in bp) distribution of transcripts in RNA-Seq libraries from pericarp and seed tissues of cotton with and without *Aspergillus flavus* infection. NIP = non-inoculated pericarp, NTP = non-toxigenic pericarp, TP = toxigenic pericarp, NIS = non-inoculated seed, NTS = non-toxigenic seed, TS = toxigenic seed.

The high quality reads from each of the six libraries from different experimental conditions were assembled independently into transcripts with a length more than 200 bp. The lengthwise distribution of transcripts of six independent libraries of cotton under different experimental conditions is shown in **Fig 1.** Trinity has a better resolving power than others in identifying alternative spliced transcript, and thus produces less duplicates and chimeric transcripts [33]. However, redundancy was encountered in the assembled transcriptome due to high sequencing depth, duplication and assembly process. Therefore, exactly duplicate transcripts were removed from all six libraries to obtain 586,569 unique transcripts (**Table 1).** Further, total transcripts from six libraries clustered into 190,558 unigenes (**Table 1)**. The size of the transcripts ranged from 201 to 15,441 bp with a mean of 1307.80 bp. Similarly, unigenes size ranged from 201 to 15,441 bp with a mean of 841.88 bp (**Table 1)**. Of the total reads, 87% aligned to the assembled transcripts indicating good coverage of the transcriptome. The high N50 and N90 values for unique transcriptome were 1864 bp and 675 bp, respectively, further suggesting a good quality assembly. Furthermore, complete alignment of the longest transcript comp54729_c0_seq1_NIP (15,441 bp) from NIP library to a gene coding for auxin transport protein of *Theobroma cacao* (TCM_019010) in the NCBI database demonstrated that the transcript was not chimeric which could have occurred due to repetitive regions in the genes. These results strongly supported a high quality transcriptome assembly of cotton.

### 3. Functional Annotation

Out of the 586,569 total unique transcripts, 466,054 (79.45%) were assigned functions based on their similarity to cotton protein database. The remaining un-annotated sequences were searched against NCBI nr and UniProtKB plant databases. Longer sequences produced significant blast hits as compared to the shorter sequences. Out of the total annotated transcripts, 405,652 (87.04%) transcripts with more than 500 bp length showed similarity to proteins in the cotton database. Of the remaining unmapped transcripts, 8,755 transcripts mapped to plant NCBI nr/nt and UniProtKB database. Further, the cotton unique transcriptome was mapped to the protein sequences of *G*. *arboreum*. In total, 19,750 un-annotated unique transcripts matched with *G*. *arboreum* protein sequences. Thus, we annotated total 494,559 transcripts (84.31%) of the cotton transcriptome. The transcripts which did not match to known genes may represent novel genes or genes that may have diverged from their homologs or noncoding RNAs [34]. The homology search showed 79.45% and 82.89% unique transcripts matching to *G*. *raimondii* and *G*. *arboreum* proteins, respectively.

### 4. Identification of Differentially Expressed Genes (DEGs) in Response to *Aspergillus flavus* Infection

**Fig 2:**
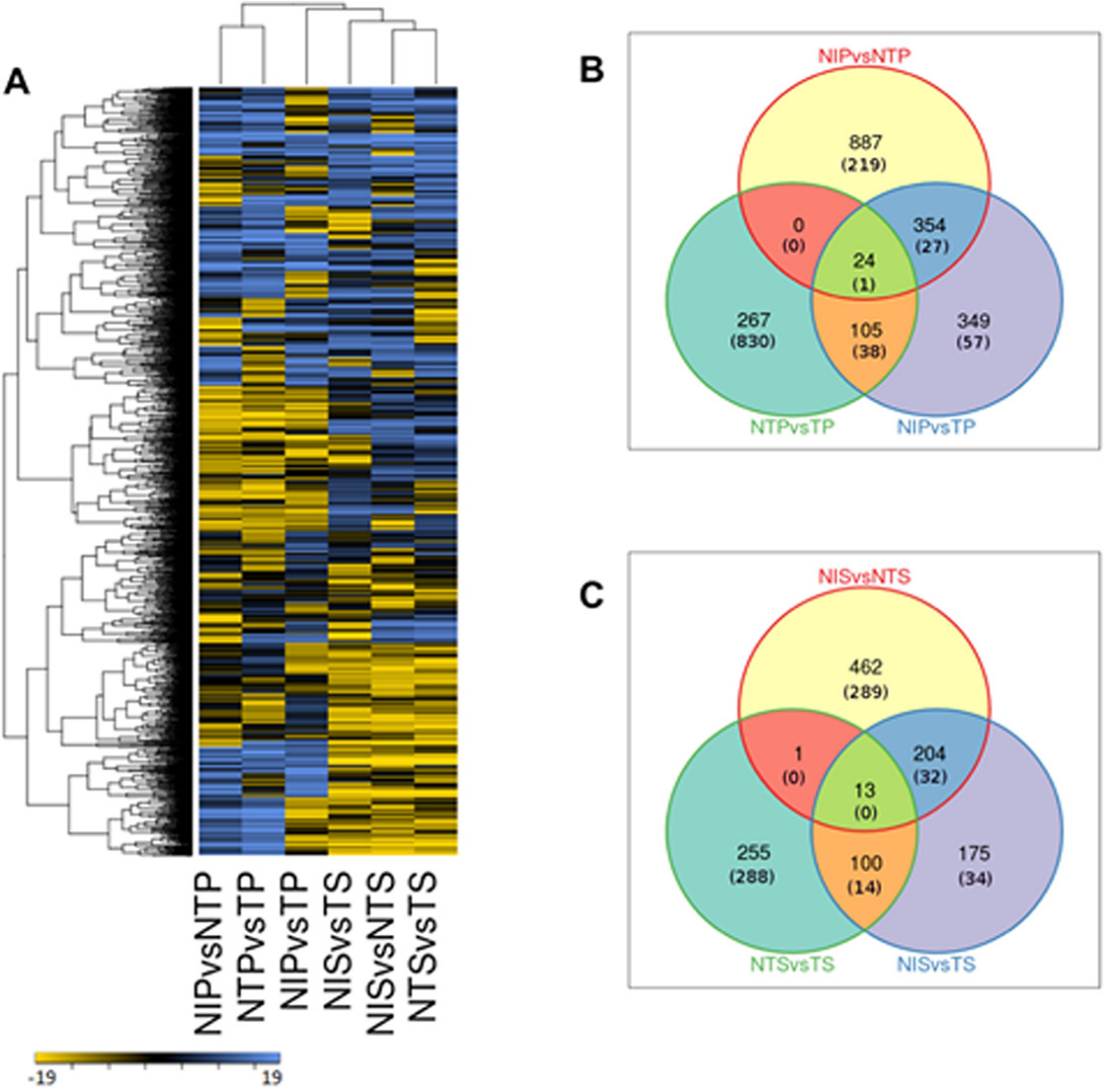
Gene expression profile of cotton in pericarp and seed tissue in response to *A*. *flavus* infection. A) Heatmap showing differentially expressed genes (DEGs) of cotton in response to infection by atoxigenic and toxigenic strains of *A*. *flavus*. The up-regulated genes (log2FC> = 2 and P<0.05) and down-regulated genes (log2FC< = -2 and P<0.05) are represented by blue and yellow color, respectively. Genes with similar expression profiles were clustered together by hierarchical clustering. For description of the gene names represented in the heatmaps please refer to the **S1 Table, sheet 2.** Venn diagram shows the unique and common DEGs in pericarp (B) and seed (C) tissues under different experimental conditions.

**Fig 3:**
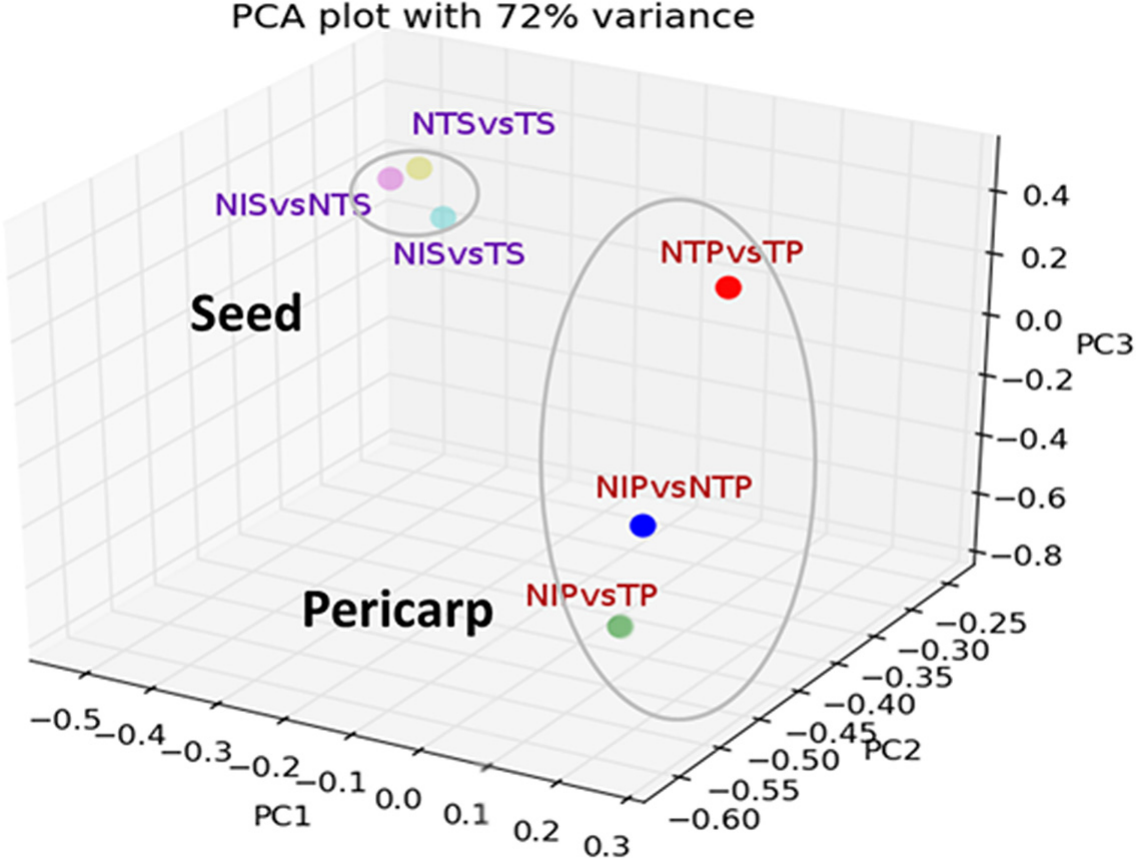
Principal component analysis (PCA) showing the variability (72% variance) of DEGs of cotton in pericarp and seed tissue in response to infection by toxigenic and atoxiganic strains of *A*. *flavus*. Expression of genes under different experimental conditions in seed (small oval) and pericarp (large oval) were distinct with the variability of expression higher in pericarp compared to seed. PC1, PC2 and PC3 explained 32%, 24% and 16% of the total variance.

Statistically significant differentially expressed genes (DEGs) in terms of FPKM (fragments per kb per million mapped reads) were calculated using combination of log2FC and P-value criteria based on mapping of the cotton reads against the *G*. *raimondii* genome as reference. In pericarp tissue, 1265, 832 and 396 genes were up-regulated (log2FC ≥2, P < 0.05) under NIP vs NTP, NIP vs TP and NTP vs TP conditions, respectively. On the other hand, 247, 123 and 869 genes were down-regulated (log2FC ≤-2, P < 0.05) under same experimental conditions, respectively. Similarly, in the seed tissue, 680, 492 and 369 genes were up-regulated under NIS vs NTS, NIS vs TS and NTS vs TS conditions, respectively, whereas, 321, 80 and 302 genes were down-regulated under same experimental conditions, respectively (**Fig 2A, 2B and 2C)**. Principal component analysis (PCA) showed distinct response of pericarp and seed tissues to toxigenic and atoxigenic strains of *A*. *flavus* (**Fig 3)**. The total variance contributed by three principal components was 72% (**Fig 3)**. The results further showed significant differences in the expression profile of genes in response to atoxigenic and toxigenic infection in pericarp, whereas, in seed, the difference in response was not significant with the infection by both strains of *A*. *flavus*. This suggested that the pericarp tissue, being the primary tissue for inoculation, exhibited higher level of differential response of genes as compared to the seed tissue. Thus, identification of specific category of highly up-regulated genes from different clusters, and characterization of their biochemical response would provide potential candidates for functional characterization through genetic manipulation toward improvement of resistance to *A*. *flavus* infection. The distributions of DEGs under different conditions are shown in three-way Venn diagrams for pericarp (**Fig 2B)** and seed **(Fig 2C)**. The DEGs were further characterized into different groups based on their putative functional significance as described below. Because *A*. *flavus* is a necrotrophic fungus, JA and ET dependent signaling pathways presumably function in defense response and regulate the expression of defense related genes, genes involved in oxidative burst, synthesis of antimicrobial compounds, regulation of transcription factors and localized programmed cell death in cotton in response to the fungal infection.

#### Genes interfering with fungal virulence and growth

Eleven transcripts encoding chitinases were differentially expressed under infection by both atoxigenic and toxigenic strains in pericarp and seed. The transcripts encoding B-CHI and CHIV were induced by infection with both atoxigenic and toxigenic strains in pericarp and seed. But, *CTL2* was up-regulated specifically in pericarp by both strains, whereas, *CHIA* was specifically induced in seed by both strains. Among the transcripts encoding CTL2, *Gorai*.*006G078900* and *Gorai*.*011G198500* were induced under atoxigenic and toxigenic strain infection, respectively. The three genes encoding β-1,3-glucanases (BG) were up-regulated specifically in seed. *BG3* (*Gorai*.*006G134600*) and *BG1* (*Gorai*.*006G134700*) were highly induced by the atoxigenic strain, whereas, another *BG3* transcript (*Gorai*.*010G003600*) was up-regulated specifically by the toxigenic strain. Plant pathogenic fungi infect the plants through wounds or release of hydrolytic enzymes, such as pectinases, proteases and amylases for successful colonization [22]. Therefore, identification and characterization of plant genes, which interfere with invasion of fungus in plants, can be useful to reduce fungal pathogenicity. The hydrolytic enzymes chitinase and β-1,3-glucanase genes possess antifungal activity by degrading the fungal cell wall containing chitins [22], [35], [36]. Plant chitinases possess lysozyme activity and are highly active in inhibiting fungal growth [37]. Moreover, over-expression of chitinase genes has conferred resistance to fungal infection in plants, such as tobacco, peanuts and rice [38–40].

**Fig 4:**
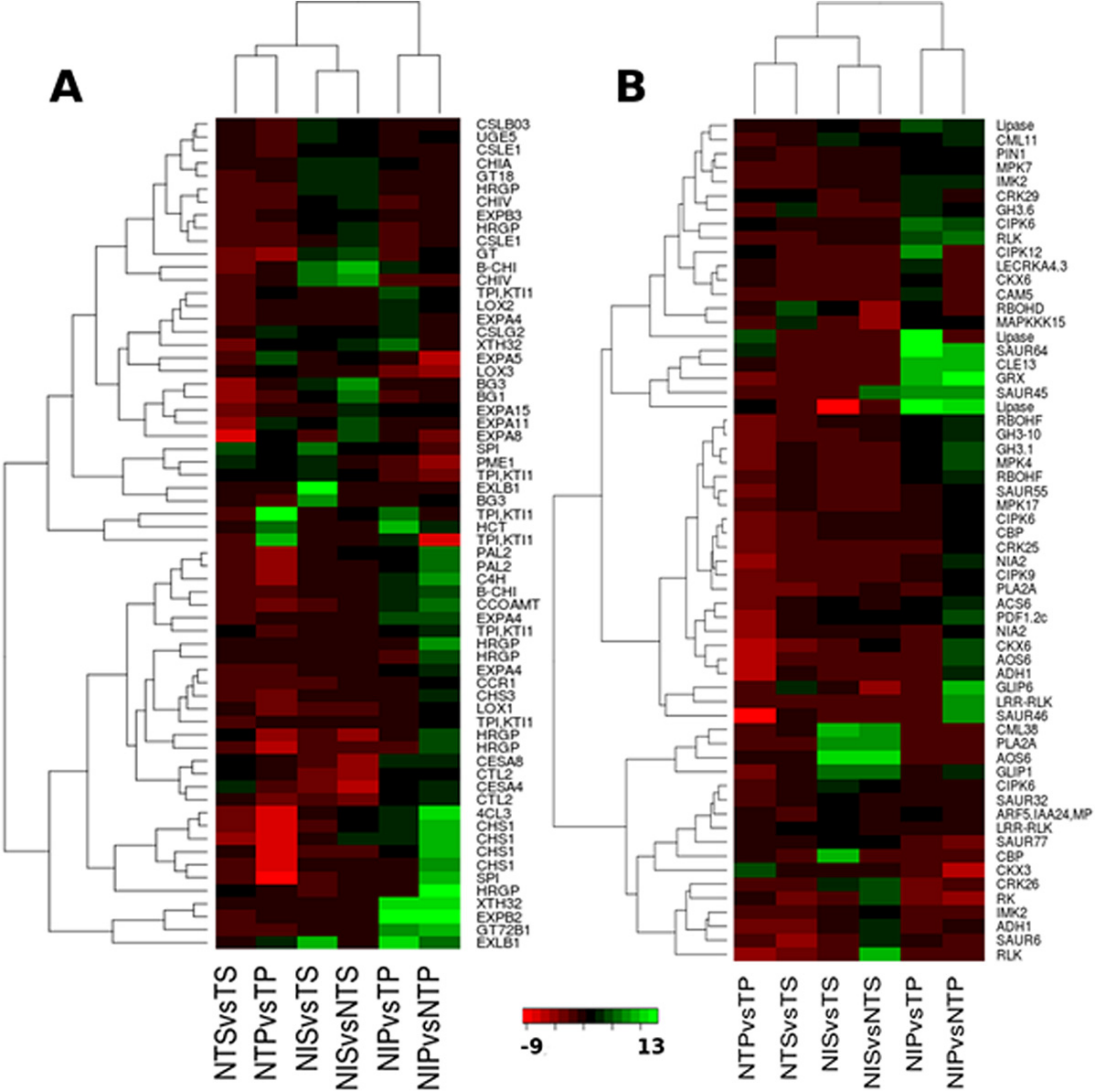
Heatmaps showing DEGs involved in interference of fungal virulence and growth (A) and DEGs involved in defense signaling (B). The green color represents up-regulated (log2FC≥2) genes and red color represents down-regulated (log2FC≤2) genes. For description of the gene names represented in the heatmaps please refer to the **S1 Table, sheet 3.**

Five transcripts encoding trypsin and protease inhibitor proteins (TPI) were induced in pericarp, and only one *TPI* was induced in seed (**Fig 4A**). The two transcripts, *Gorai*.*011G254400* and *Gorai*.*012G027700*, were up-regulated under infections by both the strains in pericarp, but the *TPI* (*Gorai*.*011G254900*) was specifically induced under the atoxigenic infection in pericarp. Among the *TPI* genes induced in pericarp, *Gorai*.*011G254500* and *Gorai*.*011G254600* were highly up-regulated by the toxigenic strain and down-regulated by the atoxigenic strain (**Fig 4A**). For example, *Gorai*.*011G254500* and *Gorai*.*011G254600* were up-regulated by 2.9- and 6.5-fold, and 9.8- and 12.7-fold higher by the toxigenic strain in comparison with the non-inoculated control and the atoxigenic strain, respectively (**S1 Table, sheet 3)**. In seed, the *TPI* (*Gorai*.*012G027600*) was induced under the toxigenic strain infection only. Reduced growth of *A*. *flavus* has direct impact on aflatoxin production [41]. Trypsin inhibitors are known to possess antifungal activity [22], [41] and inhibit conidia germination and hyphal growth of *A*. *flavus* [41]. Four genes encoding serine protease inhibitor (*SPI*) were also differentially expressed in pericarp and seed. *Gorai*.*012G105800* and *Gorai*.*007G143500* were up-regulated specifically under the atoxigenic strain infection in pericarp and the toxigenic strain infection in seed, respectively. Serine protease inhibitor gene has been shown to be induced in response to infection with *A*. *flavus* in peanut [20].

Plants accumulate hydroxyproline-rich glycoproteins (*HRGPs*), phytoalexins and lignin-like substances as a resistance mechanism in response to fungal pathogen infection [42]-[44]. The *HRGPs* and lignification are involved in fortifying the cell-wall structure of plants and contribute to the resistance to pathogen invasion [45]. Among 20 DEGs encoding *HRGP*, 10 and two genes were specifically induced in pericarp and seed, respectively (**Fig 4A**). In pericarp, most of these genes were induced under the atoxigenic strain infection. Similarly, in seed the two genes were induced by the atoxigenic strain infection. The increase in the level of *HRGP* transcripts has been observed in several plants in response to wound or pathogen infection, and is associated with higher resistance to pathogen [37]. The genes involved in the metabolism of phenylpropanoids, which serve as a source for furanocoumarin and isoflavonoid phytoalexins and lignins, were differentially expressed in the pericarp and seed tissues. The phenylpropanoid pathway precursors are also involved in the synthesis of lignin and phenolic substances, and possess antifungal activities [37], [42], [43]. The increase in cell wall lignification as a structural modification has been observed in plants for defense in response to fungal pathogen [37]. The enzymes phenylalanine ammonia-lyase (*PAL*), 4-coumarate CoA ligase (*4CL*) and chalcone synthase (*CHS*) are involved in the phenylpropanoid pathway [37], [42], [43]. Two genes encoding each *PAL2* and *4CL1* were highly up-regulated in pericarp under the atoxigenic strain infection (**Fig 4A**). The transcript encoding *4CL3* was up-regulated in both tissues, but had the higher fold change in gene expression in pericarp specifically under the atoxigenic strain infection as compared to the toxigenic strain infection. The enzyme chalcone synthase (CHS) was highly induced in pericarp (**Fig 4A**). The three genes encoding CHS (*Gorai*.*005G035100*, *Gorai*.*006G000200* and *Gorai*.*009G339300*) were specific to pericarp and showed higher expression under the atoxigenic strain infection. The two *CHS* genes (*Gorai*.*011G161200* and *Gorai*.*011G161300*) were up-regulated in both tissues under the atoxigenic infection, but only in pericarp under the toxigenic infection, The fold change for these two *CHS* genes were higher in pericarp as compared to seed (**Fig 4A**). Other genes involved in the lignin biosynthesis, such as cinnamate-4-hydroxylase (*C4H*), cinnamoyl-CoA reductase 1 (*CCR*), hydroxycinnamoyl-CoA shikimate/quinate hydroxycinnamoyl transferase (*HCT*) and caffeoyl-CoA 3-O-methyltransferase (*CCOAMT*), were specifically up-regulated in the pericarp tissue under fungal infection (**Fig 4A**). Two transcripts encoding *C4H*, one transcript encoding *CCR1* and *CCOAMT* each were highly up-regulated under the atoxigenic strain infection, while *HCT* was highly induced under the toxigenic strain infection in pericarp. Simultaneous up-regulation of *PAL*, *4CL* and *CHS* genes of the phenylpropanoid pathway suggested that these genes are coordinately regulated in response to fungal infection and that wounding and fungal infection at pericarp induced and accumulated these transcripts at a much higher level in pericarp as a part of an induced plant defense response to the invading fungus. The genes involved in the phenylpropanoid pathway are also induced in response to wounding [46]. The production of phytoalexins and accumulation of *PAL*, *CHS* and *HRGP* upon infection indicates hypersensitivity response, thus establishing immunity of uninfected distant cells [37], [42]. The increase in lignification was also shown to be associated with hypersensitive resistant reaction in wheat in response to *Puccinia graminis* f.sp. *tritici* infection [37], [47].

Lipoxygenase (*LOX*) genes of the LOX biosynthesis pathway were differentially expressed in pericarp tissue under *A*. *flavus* infection. The *LOX2* gene was up-regulated under infection by both the strains in pericarp, whereas, the *LOX1* (*Gorai*.*004G241400*) was specifically up-regulated under the atoxigenic strain infection in pericarp. The *LOX3* gene was down-regulated in the pericarp tissue under infection by both strains (**Fig 4A**). However, the *LOX* genes did not show any change in response to *A*. *flavus* infection in seed. Most volatile compounds and hydroperoxy fatty acids are the products of the LOX biosynthesis pathway [22]. These results suggested that *LOX* genes expression was more abundant in pericarp as a possible mechanism of resistance against *A*. *flavus* infection. Plant-derived volatile compounds have the capability to inhibit *A*. *flavus* growth and aflatoxin biosynthesis [22]. Previous studies have reported antifungal role of *LOX* genes in peanut, corn and soybean [20]. In human too, *LOX* genes were shown to be involved in degradation of aflatoxin B1 by oxidative metabolism [20].

Wounding and pathogen infection have also been shown to regulate the genes involved in cell wall biosynthesis and modifications, such as pectins, cellulose and hemicellulose [46]. The transcripts encoding xyloglucan endotransglucosylase (XTH), cellulose synthase (CS), UDP-D-galactose 4-epimerase (UGE), pectin methylesterase (PME), expansin (EXP) and glycosyltransferase (GT), which are involved in the cell wall modification, were regulated by *A*. *flavus* infection in both pericarp and seed (**Fig4A**). The results also suggested that cell-wall modifying genes and genes involved in the production of antimicrobial substances were more active in pericarp as compared to seed. It is thus evident that the atoxigenic strain of *A*. *flavus* played a major role in activation of the antifungal and cell-wall modifying genes in the pericarp and seed tissues. Further, the characteristic response of these genes under specific fungal strain infection and tissue will help elucidate the mechanism of defense.

#### The cross-talk between JA/ET and SA signaling pathway

Salicylic acid (SA), jasmonic acid (JA), ethylene (ET) and abscisic acid (ABA) are known to be involved in defense signaling pathways in response to pathogen infections and wounding [16], [17], [46], [48], [49]. SA-induced defense response generally involves resistance to biotrophic and hemibiotrophic pathogens, whereas JA and ET initiate the defense reaction in response to necrotrophic pathogens [16], [17]. The activation of SA and JA/ET pathways are pathogen dependent that involves mutually antagonistic activities [16]. JA and ET work synergistically in response to pathogen infection in plants [16]. In the present study, the transcripts encoding phospholipase, GDSL lipase, allene oxide synthase (*AOS*) and alcohol dehydrogenase (*ADH*), which are involved in the JA biosynthesis [46], [49], were induced in both pericarp and seed tissues (**Fig 4B**). Similarly, the gene 1-aminocyclopropane-1-carboxylic acid synthase (*ACS*), involved in ET biosynthesis [46], was up-regulated in both pericarp and seed tissues (**Fig 4B**). A higher number of lipase-encoding transcripts were induced in pericarp as compared to seed (**Fig 4B**). In pericarp, the lipases genes showed similar expression pattern under both atoxigenic and toxigenic strains infection, whereas in seed the genes were more active under the toxigenic strain infection (**Fig 4B**). *AOS* and *ACS6* genes were up-regulated under the atoxigenic strain infection in pericarp, while in seed *AOS* was up-regulated by both strains, and *ACS6* was specific to toxigenic strain infection (**Fig 4B**). The jasmonate zim-domain protein (*JAZ*) inhibits the JA signaling in plants by interacting with *JIN1/MYC2* gene and represses the expression of JA responsive genes [16]. The transcript encoding JAZ8 (*Gorai*.*006G092400*) was down-regulated in both pericarp and seed tissues under *A*. *flavus* infection (**Fig 4B**). Further, SA mediated signaling pathway was inhibited by JA/ET signaling pathway in both pericarp and seed tissue in response to the fungal infection. The non-expresser of PR genes (*NPR*) which are a vital component of SA signaling pathway [16] was down-regulated in both pericarp and seed in response to *A*. *flavus* infection (**S1 Table**). The mitogen activated protein kinase gene, *MPK4*, acts as a positive regulator of JA and a negative regulator of SA signaling pathway in plants [16]. *MPK4* was up-regulated under the atoxigenic strain infection in pericarp. Interestingly, the glutaredoxin (*GRX*), which is identified as a negative regulator of JA/ET signaling pathway [16], was up-regulated in pericarp, but down-regulated in seed. The enhanced expression of JA-responsive marker gene plant defensin 1.2 (*PDF1*.*2*) is known to be associated with resistance to necrotrophic pathogens [16]. In the present study, the transcript encoding PDF1.2c was specifically up-regulated under the atoxigenic *A*. *flavus* strain infection in pericarp tissue (**Fig 4B**). The transcription factor *ERF1* that acts as a positive regulator of JA and ET signaling pathway in Arabidopsis [16] was highly induced in seed by the infection with both strains of *A*. *flavus*. These results indicated that JA/ET signaling pathway may be a component in the resistance mechanism of cotton to necrotrophic *A*. *flavus*.

#### Genes involved in defense signaling pathways

Plant receptor protein kinases (*RPK*) represent PRRs that are involved in the perception of pathogen signal and trigger inducible defense [50]. The transcripts similar to receptor-like protein kinase (*RLK*), leucine-rich repeat receptor-like protein kinase (*LRR-RLK*), cysteine-rich RLK (*CRK*), lectin receptor kinase, inflorescence meristem receptor-like kinase (*IMK*) and receptor kinase (*RK*) were differentially expressed in both tissues under the atoxigenic and the toxigenic A. *flavus* strains infection (**Fig 4B**). In seed, the transcripts encoding RLK were highly induced by the atoxigenic strain. The transcripts for LRR-RLK were specifically induced by the atoxigenic strain in pericarp and the toxigenic strain in seed. The *CRK* genes were induced in both tissues by both atoxigenic and toxigenic strains (**Fig 4B**). Plants produce elicitors that are perceived by *RPK* to amplify immunity and resistance to fungal infection [51], [52]. The elicitor CLAVATA3 (*CLV3*), secreted by shoot apical meristem, binds to the LRR-RLK and activates it [51]. In pericarp, the transcript encoding CLAVATA3 (*CLV3*) was up-regulated under both atoxigenic and toxigenic strains infection (**Fig 4B**).

The increase in the level of Ca^2+^ is indicative of the activation of plant’s innate immunity. The increase in Ca^2+^ levels is the result of release of pathogen elicitors after the infection in plants. The elevated levels of calcium under stress conditions are recognized by calcium binding proteins such as calcium dependent kinases (CDPKs), calmodulins (CaMs) and calcineurin B-like proteins (CBL), which in turn induce downstream target gene expression [46], [51] [53]. The up-regulation of a large number of transcripts encoding calcium binding proteins including CaMs, CBL and calcium binding EF-hand family proteins (CBP) in the present study suggested that there were elevated levels of Ca^2+^ after fungal infection in both pericarp and seed tissues (**Fig 4B**). In pericarp, most of the transcripts encoding CBP were induced under the atoxigenic strain infection, whereas in seed these were under the toxigenic strain infection (**Fig 4B**). Recent studies on plant-pathogen interactions reported that Ca^2+-^mediated activation of CaMs, CBL interacting protein kinases (CIPKs) and CDPKs are involved in plant’s immunity responses [51]. The transcripts similar to *CIPK9*, *CIPK12*, *CIPK6* and *CIPK21* were differentially expressed in both tissues under fungal infection (**Fig 4B**). *CIPK9 and CIPK6*, and *CIPK12 and CIPK6* genes were induced under the atoxigenic and the toxigenic infection in pericarp, respectively (**Fig 4B**). However in seed, only *CIPK6* was induced under the toxigenic infection. The *CIPK* genes are involved in late immune responses (3–24 h after infection) and promote accumulation of phytoalexin, and expression of cell death and *PR* genes in response to fungal infection [51].

The activation of MAPK pathway is also one of the defense responses that contribute to resistance to fungal infection in plants starting as early as 1 min after infection. Wounding and pathogen elicitors induce fast activation of MAPK cascade signaling [51]. The MAPK cascade signaling involves three components: MAPK kinase kinase (MAPKKK), MAPK kinase (MAPKK) and MAPK. In MAPK signaling cascade, the MAPKKK activates MAPKK, which in turn activates MAPK. The four transcripts encoding MAPKKK15, MPK17, MPK7 and MPK4 were highly up-regulated specifically under the atoxigenic strain infection in pericarp (**Fig 4B**). MAPK cascade signaling was not induced in seed tissue in response to the fungal infection. The activation of MAPK signaling cascade regulates the downstream transcription factors, which further induce the expression of defense related genes leading to enhanced long-term defense response and resistance to fungal infection by regulating the synthesis of antimicrobial peptides and chemicals, programmed cell death, stress hormones (JA and ET), nitric oxide (NO) and reactive oxygen species (ROS) [51], [52]. The NO synthesis in plants is catalyzed by the enzyme nitrate reductase (NIA), which plays an important role in plant defense responses [54], [55]. Under the atoxigenic strain infection, the two transcripts encoding NIA2 were up-regulated in pericarp (**Fig 4B**). In tobacco cells, the fungal elicitors contributed to prolonged activation of MAPKs, which regulate the expression of NO and ROS [51], [56]. The ROS, which play an important role in plant defense responses together with NO, are synthesized by the phagocyte enzyme complex of NADPH oxidase [54], [56]. The respiratory burst oxidase homolog (*RBOH*) which is plant NADPH oxidase [56], [57] was differentially expressed in both pericarp and seed tissues of cotton under fungal infection (**Fig 4B**). The *RBOH* is regulated by MAPK signaling cascade and its increased expression is associated with resistance to pathogens [51], [56]. The three homologs of *RBOH* including *RBOHF* (*Gorai*.*003G085100*, *Gorai*.*008G199100*) and *RBOHD* (*Gorai*.*009G202500*) were highly induced specifically in pericarp (**Fig 4B**). The *RBOHF* transcripts were induced under both atoxigenic and toxigenic strains infection in pericarp, whereas *RBOHD* was specifically up-regulated under the toxigenic strain infection (**Fig 4B**). The production of NO and ROS together are necessary for inducing the hypersensitive response (HR) and cell death in plants [54].

The phytohormone auxin (Aux), besides regulating growth and developments of plants, plays an important role in the defense responses to pathogens, [16], [58], [59]. Aux regulates the expression of *Aux/IAA*, Gretchenhagen-3 (*GH3*) family, Auxin response factor (*ARF*) and small auxin-up RNA (*SAUR*) genes [16], [58], [60]. Over-expression of *OsGH3*.*1* in rice enhanced the resistance to fungal pathogen by reducing the auxin level and suppressing the expression of expansin genes [16], [59]. In this study, the transcripts similar to *GH3*.*1* and *GH3*.*10* were specifically induced in pericarp by both strains ns (**Fig 4B**). The *GH3*.6 (*Gorai*.*005G208000*) was specifically induced under the toxigenic strain infection in pericarp. Similarly, *SAUR* family genes were differentially expressed in pericarp and seed tissues under *A*. *flavus* infection (**Fig 4B**). *SAUR* genes have inhibitory activity on auxin biosynthesis and transport [60]. The over-expression of *SAUR39* transcript in rice showed reduced free IAA level and auxin transport [60].

The role of phytohormone cytokinin has also been elucidated in disease resistance reaction in *Arabidopsis* [16], [61]. *Arabidopsis* lines overexpressing cytokinin oxidase/dehydrogenase (*CKX*) showed enhanced resistance to clubroot disease [61]. The transcripts similar to *CKX6* (*Gorai*.*012G081000* and *Gorai*.*011G295400*) were induced under atoxigenic and toxigenic strains infection only in pericarp. Contrastingly, *CKX3* was down-regulated in pericarp under the atoxigenic strain infection (**Fig 4B**). The detailed characterization of these genes is necessary to understand the role and cross-talk of auxin- and cytokinin-signaling pathways in *A*. *flavus*-mediated defense response in cotton.

#### Genes encoding transcription factors (TFs)

**Fig 5:**
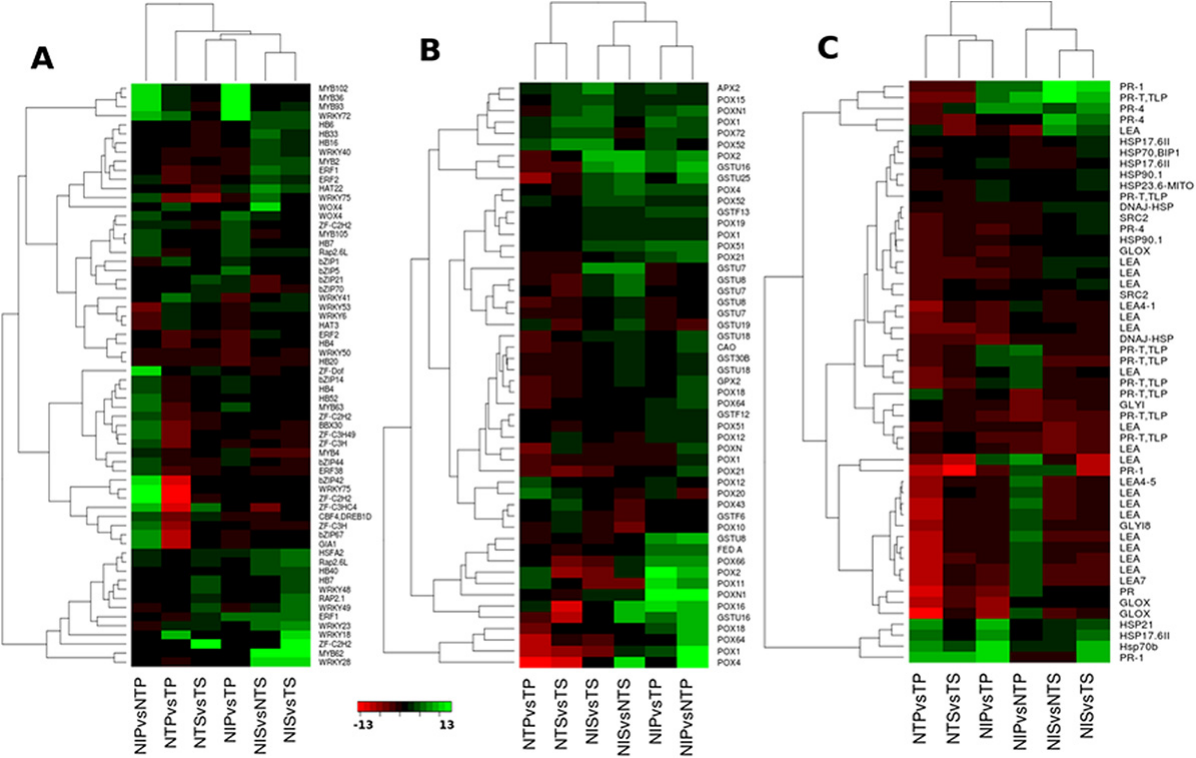
Heatmaps showing DEGs involved in transcriptional regulation (A), involved in oxidative burst (B) and stress response (C). The green color represents up-regulated (log2FC≥2) genes and red color represents down-regulated (log2FC≤2) genes. For gene names represented in the heatmaps please refer to the **S1 Table**.

Transcription factors control the transcriptional regulation by activating or suppressing the expression of downstream genes in response to pathogens infection [62]. The transcription factor *GhWRKY3* is known to be induced under wounding and fungal infection in cotton [62]. Mutation in *WRKY70* and *WRKY33* enhanced the susceptibility of *Arabidopsis* to necrotrophic *B*. *cinerea* fungal infection [17]. *WRKY40*, *WRKY33*, *WRKY53*, *WRKY22*, *WRKY11*, *WRKY15* and *WRKY60* are known to be induced under wounding in *Arabidopsis* [46]. In the present study, 28 WRKY-related transcripts were differentially expressed under fungal infection in pericarp and seed of cotton **(Fig 5A)**. The *WRKY75* (*Gorai*.*001G057600* and *Gorai*.*005G164300*) and *WRKY72* were induced in both pericarp and seed in response to *A*. *flavus* infection. Most of the WRKY TFs were up-regulated under the atoxigenic strain infection in pericarp and the toxigenic strain infection in seed **(Fig 5A)**. *WRKY75* (*Gorai*.*006G043200*) and *WRKY40* (*Gorai*.*009G124000*) were specifically up-regulated under the atoxigenic strain infection in both pericarp and seed. *WRKY6* (*Gorai*.*001G214800)*, *WRKY53* (*Gorai*.*008G253300)*, *WRKY50* (*Gorai*.*001G021500*) and *WRKY41* (*Gorai*.*007G014600*) were down-regulated in pericarp. *WRKY50* is reported to negatively regulate the plant-fungus interaction, and mutation in *WRKY50* has been associated with enhanced resistance to pathogen [17]. It was also reported that the *WRKY* TFs regulate the expression of *RLK* genes, which are induced in response to pathogen infection [46], [63].

The APETALA2 (AP2)/Ethylene responsive factor (ERF) family of TFs are known to be involved in the activation of defense-related genes in response to pathogen infection through ET and JA pathways [17], [64]. The members of AP2/ERF family were differentially expressed in response to the necrotrophic *A*. *flavus* infection in pericarp and seed **(Fig 5A)**. The transcripts similar to *RAP2*.*6L* (*Gorai*.*012G125700*) and *ERF38* were up-regulated specifically in pericarp **(Fig 5A)**. *RAP2*.*6L* was induced under both atoxigenic and toxigenic strains infection, whereas, *ERF38* expression was specific to the atoxigenic strain infection. The number of *AP2/ERF* TFs up-regulated in seed was higher as compared to pericarp. *ERF1* (*Gorai*.*005G049300*) and *RAP2*.*6L* (*Gorai*.*005G197100)* were induced specifically in seed under both strain infections. In seed, *ERF2* (*Gorai*.*009G165500)* and *RAP2*.*1* were up-regulated under the atoxigenic and the toxigenic strain infection, respectively. However, *ERF2* (*Gorai*.*010G156600)* was down-regulated under the toxigenic strain infection in pericarp. The *AP2/ERF* TF family genes are known to be highly induced in response to wounding in *Arabidopsis* [46]. Overexpression of an *ERF* TF in *Arabidopsis* conferred enhanced resistance to necrotrophic fungus *B*. *cinerea* [17]. A transcript encoding *DREB1D/CBF4* gene was induced under the atoxigenic strain infection in pericarp **(Fig 5A)**. *DREB/CBF* TFs belong to *AP2/ERF* TF family and have been reported to be induced in response to wounding, in addition to abiotic stresses, such as cold, heat, salt and drought in *Arabidopsis* [46].

The role of bZIP family TFs in biotic stress responses of plants is well established [65]. The bZIP-family TFs were induced in pericarp of cotton by *A*. *flavus* infection. The five bZIP TFs, *bZIP14*, *GIA1*, *bZIP67*, *bZIP44* and *bZIP42*, were specifically induced under the atoxigenic strain infection, whereas *bZIP1* and *bZIP5* were induced under the toxigenic strain infection **(Fig 5A)**. The bZIP TFs were not induced by the fungus in seed; *bZIP70* and *bZIP21* were down-regulated under the atoxigenic strain infection in seed.

Many *MYB* TFs were also differentially expressed under *A*. *flavus* infection in cotton. More MYB genes were up-regulated in pericarp as compared to seed **(Fig 5A)**. *MYB93*, *MYB63*, *MYB102*, *MYB105*, *MYB102* and *MYB36* were induced under both the atoxigenic and the toxigenic strains infection in pericarp, whereas *MYB62* and *MYB2* were induced in seed **(Fig 5A)**. The gene expression pattern of *MYB* TFs has been studied in *Arabidopsis* in response to wounding and pathogen infection [46]. The *MYB* TFs regulate the expression of flavonoid genes, *PR* genes and genes involved in secondary metabolism [46]. In the present study, *MYB4*, which is a repressor of phenylpropanoid pathway [49], was down-regulated under both strains infection in seed **(Fig 5A).**

The gene expression profiles of zinc finger (ZF), heat shock factors (HSF) and homeobox (HB) type TFs were altered in response to wounding in *Arabidopsis* [46]. In cotton, a number of transcripts encoding *ZF* TFs were up-regulated in response to the atoxigenic strain infection in pericarp, whereas in seed, only one transcript similar to *ZF-C2H2* (*Gorai*.*002G223300)* was induced under the toxigenic strain infection **(Fig 5A)**. The HB type TFs showed high activity in seed than pericarp, which was evident from the up-regulation of a large number of *HB* genes in seed as compared to pericarp **(Fig 5A)**. The *HB* TFs, *HAT3*, *HB20* and *HB4*, were down-regulated in pericarp under *A*. *flavus* infection. The HSFs did not show any activity in pericarp, and were specifically induced in seed **(Fig 5A)**. The transcript showing similarity with *HSFA2* was highly up-regulated in seed under both strains infections. Further characterization of these TFs is necessary to understand their regulatory mechanisms in controlling expression of downstream genes and their roles in defense response of cotton to *A*. *flavus* infection.

#### Genes involved in oxidative burst

Oxidative burst is one of the earliest defense responses of plants against pathogen infection and wounding, and is considered as the hallmark of pathogen recognition [46], [56], [57], [66]. ROS production is observed in both plant triggered immunity (PTI) and effector triggered immunity ETI [57]. In addition to their involvement in direct defense reaction by killing the pathogens, ROS are also involved in the activation of defense-related genes through signaling mechanism [57]. ROS can regulate the TFs and produce antimicrobial phytoalexins and other secondary metabolites, which have inhibitory activity on pathogen growth [57]. As discussed earlier, the MAPK pathway activated the expression of *RBOH* genes in response to *A*. *flavus* infection, which could trigger the plant apoplastic oxidative burst. The transcripts encoding glutathione S-transferase (GST), ascorbate peroxidase (APX), copper amine oxidase (CAO), ferredoxin (FED) and peroxidase (POX), which are involved in ROS processing and scavenging, showed increased activity under *A*. *flavus* infection in cotton (**Fig 5B**). Most of these genes were induced in pericarp, and their expression patterns were different under the atoxigenic and the toxigenic strains infection in both pericarp and seed tissues. The transcripts similar to copper amine oxidase (*CAO*) under atoxigenic strain infection and ferredoxin under both strains infections were specifically induced in pericarp tissue (**Fig 5B**). Most of the transcripts encoding glutathione S-transferase (GST) were induced under the atoxigenic strain infection in both pericarp and seed (**Fig 5B**). The expression patterns for peroxidase genes (*POX*) were similar under the atoxigenic and the toxigenic strain infection in pericarp, but in seed *POX* genes were highly induced under the toxigenic strain infection (**Fig 5B**). The peroxidase 2 (*POX2*) genes were specifically induced in pericarp tissue under both the atoxigenic and the toxigenic strains infection. The transcript similar to glutathione peroxidase (*GPX2*) was induced under the atoxigenic strain infection in pericarp (**Fig 5B**). Hydrogen peroxide can also act as a secondary messenger and initiate regulation of defense related genes [46] These results suggested that wounding and subsequent *A*. *flavus* infection activated the ROS-regulated defense response in both pericarp and seed tissues of cotton.

#### Genes involved in stress response

Many stress responsive genes have been implicated in plant’s response to fungal infection [4], [22]. The late embryogenesis abundant (*LEA*) storage protein was shown to be induced in response to *A*. *flavus* infection in cotton [4]. In this study, 12 transcripts similar to *LEA* genes were specifically up-regulated in response to the atoxigenic *A*. *flavus* infection in pericarp (**Fig 5C**). In seed, only 4 *LEA4* transcripts were differentially induced under the atoxigenic and the toxigenic strains infection. The heat shock proteins (HSP) play a major protective role in biotic and abiotic stresses by controlling chaperone activity and other cellular processes [22], [46]. The expression of *HSPs* is under the regulation of *HSFs* [46]. There are several *HSPs* and *HSFs* that have been reported to be induced by pathogen infection and wounding [22], [46]. Most of the transcripts similar to *HSPs*, in this study, were induced in seed as compared to pericarp tissue (**Fig 5C**). *HSP70B* was specifically induced under the atoxigenic strain infection in pericarp. *HSP23*.*6-MITO*, *HSP90*.*1*, *HSP21* and *HSP17*.*6II* were all induced in both pericarp and seed tissues by both the strains. *HSP90*.*1* and *DNAJ-HSP* were up-regulated byboth the strains specifically in seed, whereas *HSP70* was up-regulated under the toxigenic strain infection. The *DNAJ-HSP* (*Gorai*.*008G099300*) was down-regulated in pericarp tissue. The up-regulation of a large number of *HSPs* in seed as compared to pericarp could be correlated to the induction of *HSFs* specifically in seed tissue, as discussed earlier. Stress-inducible cold regulated gene (*SRC2*) was specifically up-regulated in the seed tissue (**Fig 5C**). Two transcripts encoding glyoxal oxidase-related protein were specifically induced under the toxigenic strain infection in seed (**Fig 5C**). The overexpression of glyoxal oxidase gene was shown to enhance the resistance of grape plant to fungal infection [67]. The stress responsive gene glyoxalase I was also known to be induced in response to abiotic and biotic stresses in plants [22], [68]. Glyoxalase I was up-regulated in response to necrotrophic hemibiotroph fungus *T*. *basicola* in *G*. *hirsutum* [68]. The expression of a transcript coding for a glyoxalase I family protein, GLYI8, was specific to pericarp and up-regulated in response to the atoxigenic *A*. *flavus* infection (**Fig 5C**). The pathogenesis related genes (*PR*) that are associated with the resistance reactions [22], [68], [69] were also differentially expressed in the pericarp and seed tissues (**Fig 5C**). Among the 15 differentially expressed transcripts that were similar to *PR* genes, seven and three were specific to pericarp and seed, respectively. In pericarp, *PR* and *PR-1* (*Gorai*.*006G115900*) were up-regulated specifically under the atoxigenic strain infection, and *PR-T* (*Gorai*.*007G193600*) was up-regulated under the toxigenic strain infection. Two PR genes (*PR-4* and *PR-1*) were equally induced in seed, while *PR-T* (*Gorai*.*009G194000*) was down-regulated under both strains infection in seed. Most of these stress responsive genes have also been known to be induced under abiotic stress conditions.

### 5. GO and KEGG Enrichment Analysis of DEGs

**Fig 6:**
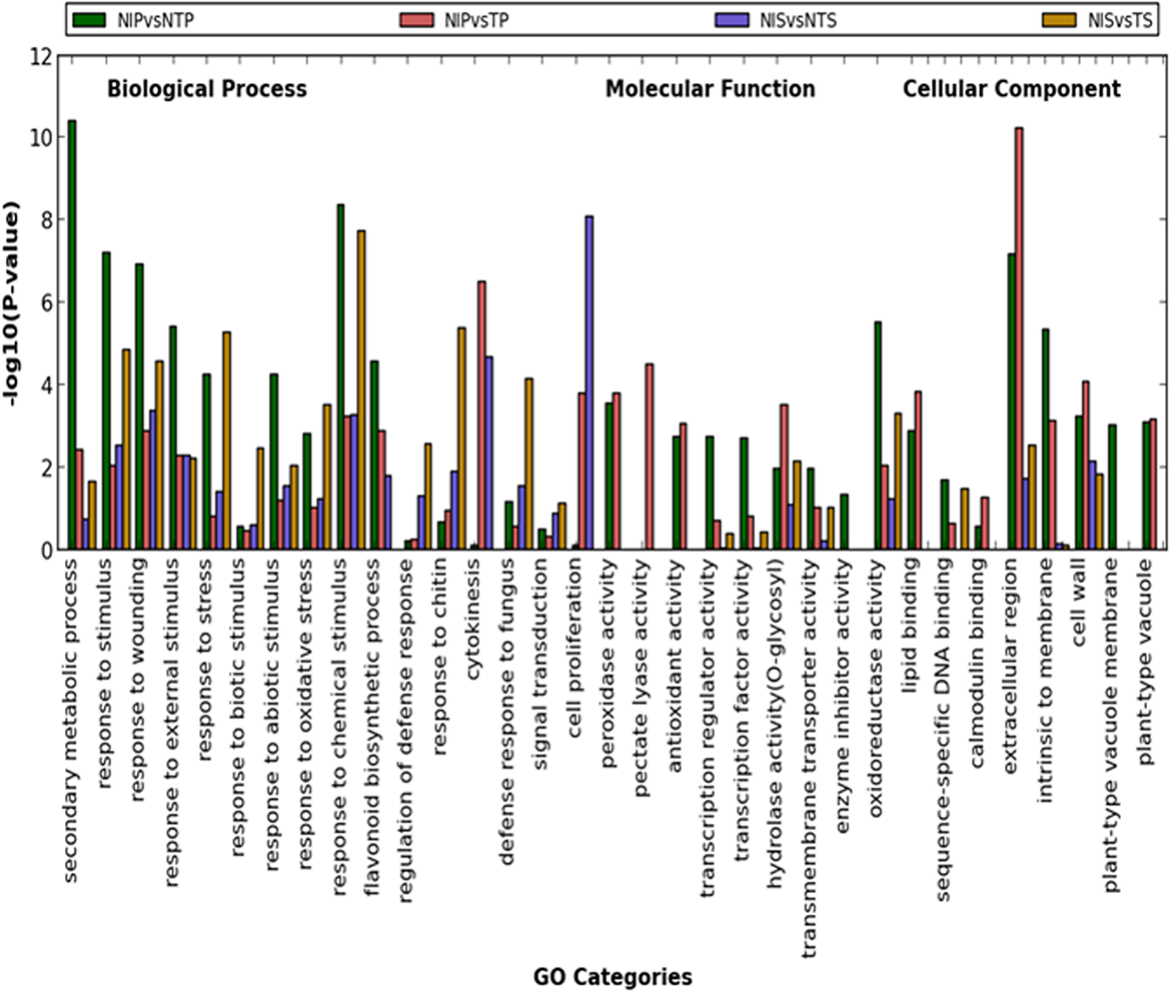
Gene ontology enrichment analysis of DEGs in pericarp and seed tissues of cotton in response to infection with atoxigenic and toxigenic strains of *A*. *flavus*. The X-axis represents the GO categories and Y-axis represents enrichment in terms of P-value.

In this study, GO enrichment analysis of the DEGs identified the functional categories, such as biological process, molecular function and cellular component that were distinctly represented by the atoxigenic and the toxigenic strain infection in pericarp and seed tissues of cotton. The GO categories under biological process, such as response to stimulus (GO:0050896), response to wounding (GO:0009611), response to external stimulus (GO:0009605), response to chemical stimulus (GO:0042221) and flavonoid biosynthesis (GO:0009813) were highly represented under the atoxigenic strain infection in pericarp. On the other hand, response to biotic stimulus (GO:0009607), response to stress (GO:0006950), regulation of defense response (GO:0031347), response to chitin (GO:0010200), defense response to fungus (GO:0050832) and signal transduction (GO:0007165) were enriched under the toxigenic strain infection in seed (**Fig 6)**. Under molecular function category, most of the responses were highly enriched in pericarp as compared to seed. In pericarp, transcription regulator activity (GO:0030528), transcription factor activity (GO:0003700), transmembrane transporter activity (GO:0022857) and enzyme inhibitor activity (GO:0004857) were enriched under the atoxigenic strain infection in comparison with the toxigenic strain infection (**Fig 6)**. Peroxidase activity (GO:0004601), pectate lyase activity (GO:0030570), antioxidant activity (GO: 0016209) and hydrolase activity (GO:0004553) were enriched under the toxigenic strain infection in pericarp as compared to the atoxigenic strain infection (**Fig 6)**. Most of the cellular components were enriched in pericarp as compared to seed tissue (**Fig 6).** Component of cell wall, membrane and vacuoles were differentially enriched under the atoxigenic and the toxigenic strain infection in pericarp and seed.

**Fig 7:**
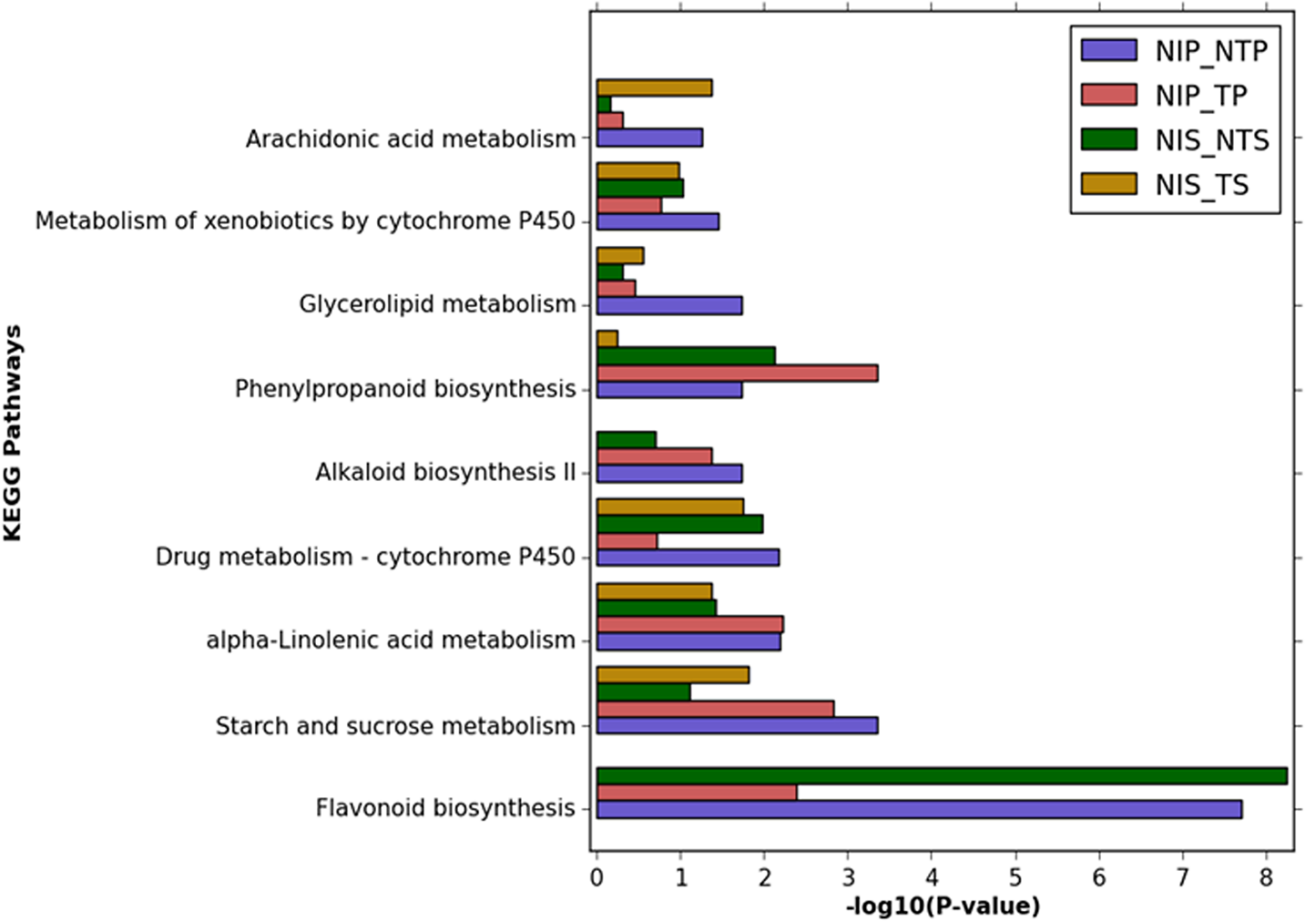
Highly represented KEGG metabolic pathways in pericarp and seed tissues of cotton under *A*. *flavus* infection. The X-axis represents the enrichment in terms of P-value and Y-axis represents the biochemical pathways.

Analysis of the biochemical pathways represented by the DEGs showed that 94, 77, 59, and 63 KEGG pathways were represented under the atoxigenic and the toxigenic strains infection in pericarp and seed, respectively. Highly enriched pathways (P < 0.05) in pericarp and seed are shown in **Fig 7**. The phenylpropanoid pathway, which is involved in the production of antimicrobial phytoalexins, lignins and phenolic substances [37], [42], was enriched in the toxigenic strain infection in pericarp followed by the atoxigenic infection in pericarp and seed. The flavonoid biosynthesis pathway was the most highly enriched under the atoxigenic strain infection in seed followed by pericarp. Genes in the flavonoid pathway are involved in the production of antifungal compounds and are associated with defense reactions [70]. The alkaloid biosynthesis pathway was highly enriched under both atoxigenic and toxigenic strains infection in pericarp as compared to seed (**Fig 7)**. In tobacco plants, alkaloid biosynthesis is induced in response to insect damage and application of jasmonate [71]. This suggests that JA-regulated defense response was activated in cotton in response to *A*. *flavus* infection. Further, enrichment of arachidonic acid (AA) metabolism was observed under the toxigenic strain infection in seed followed by the atoxigenic infection in pericarp (**Fig 7)**. AA acts as a signaling molecule, and activates plant’s defense responses through fatty acids. AA is a potent elicitor present in the pathogen, which activates plant innate immunity leading to programmed cell death and defense responses [72]. The alpha-linolenic acid metabolism pathway was enriched in pericarp in comparison to seed under both atoxigenic and toxigenic strains infection (**Fig 7).** JA and its derivatives, which are key regulators of plant defense responses to necrotrophic pathogens, are synthesized from the alpha-linolenic acid pathway [73], [74]. The primary metabolic pathways, such as starch and sucrose metabolism and glycerolipid metabolism, were also highly enriched under the atoxigenic strain infection in pericarp (**Fig 7)**. The up-regulation of carbohydrate, amino acids and lipid metabolisms was suggested to regulate the signal transduction cascade during plant defense responses [75]. The biochemical pathways involved in response to the atoxigenic strain of *A*. *flavus* infection in pericarp and seed tissue of cotton can be manipulated for stress tolerance in cotton.

Validity of the next generation sequence data was confirmed by reverse-transcription PCR of 10 genes belonging to different functional categories with fold change expression of 5-fold or above (from sequence data) under experimental conditions relative to non-inoculated control. The results showed significant up-regulation of their mRNA accumulation under infection by the atoxigenic or the toxigenic strain infection in a tissue-dependent manner (**S1 Fig**).

## Conclusions

Little is known about the molecular mechanisms of *A*. *flavus* resistance in cotton, largely due to the lack of a resistant cotton germplasm. However, understanding the expression profile of the genes, especially in response to the atoxigenic strain infection, could provide clues to the molecular mechanism of resistance, in addition to the physical barrier, conferred by the atoxigenic strains against the toxigenic strain. Comparative analysis of the genes involved in specific GO categories of the atoxigenic vis-à-vis the toxigenic strain infection will lead to the identification of promising candidates for genetic manipulation toward development of cotton varieties resistant to *A*. *flavus*. For example, genes with transcriptional regulation involved in response to stress stimulus, involved in flavonoid biosynthesis and lipid biding in extracellular regions (Fig 6) could be considered promising candidates for further validation through functional characterization. In addition, our ongoing comparative analysis of the cotton transcriptome with available corn and peanut transcriptome, induced under *A*. *flavus* infection, will provide a better understanding of the genetic and biochemical basis of *A*. *flavus*-cotton interaction and also identify conserved orthologous genes in cotton for their functional translation in conferring resistance to *A*. *flavus* through genetic manipulation in cotton.

## Supporting Information

S1 Fig Gel image (upper panel) showing semiquantitative RT-PCR and fold-change expression through quantitative RT-PCR (lower panel) of genes under infection by atoxigenic and toxigenic strains of *Aspergillus flavus* in pericarp and seed tissues of cotton. Gene expression was normalized using cotton elongation factor [4] as the internal reference against the expression in non-inoculated control, which was set to 1.
(TIF)

S1 Table Details of differentially expressed genes under infection by atoxigenic and toxigenic strains of *Aspergillus flavus* in seed and pericarp tissues of cotton. Sheet 1, Nomenclature of genes and primer sequences used for expression analysis through RT-PCR; sheet 2, All differentially expressed genes discussed in the manuscript and used for heatmap in Fig 2A; sheet 3, Genes from different classes used in the generation of heatmaps for Figs 4 and 5.
(XLSX)

## References

[1] Cleveland TE, Dowd PF, Desjardins AE, Bhatnagar D, Cotty PJ. United States Department of Agriculture—Agricultural Research Service research on pre-harvest prevention of mycotoxins and mycotoxigenic fungi in US crops. Pest Manag Sci. 2003;59(6–7):629–42. 12846313 10.1002/ps.724

[2] Wogan GN. Impacts of chemicals on liver cancer risk. Semin Cancer Biol. 2000;10(3):201–10. 10936069 10.1006/scbi.2000.0320

[3] International Agency for Research on Cancer (IARC). IARC Monographs on the evaluation of the carcinogenic risks to humans: some naturally occurring substances: food items and constituents, heterocyclic aromatic amines and mycotoxins IARC, Lyon, France 1993;56:245–540.

[4] Lee S, Rajasekaran K, Ramanarao MV, Bedre R, Bhatnagar D, Baisakh N. Identifying cotton (*Gossypium hirsutum* L.) genes induced in response to *Aspergillus flavus* infection. Physiol Mol Plant Pathol. 2012;80:35–40.

[5] Yin YN, Yan LY, Jiang JH, Ma ZH. Biological control of aflatoxin contamination of crops. J Zhejiang Univ-Sc B. 2008;9(10):787–92. 10.1631/jzus.B0860003 PMC2565741 18837105

[6] Kelley RY, Williams WP, Mylroie JE, Boykin DL, Harper JW, Windham GL, et al Identification of Maize Genes Associated with Host Plant Resistance or Susceptibility to *Aspergillus flavus* Infection and Aflatoxin Accumulation. Plos One. 2012;7(5). 10.1371/journal.pone.0036892 PMC3351445 22606305

[7] Richard JL, Payne GA. Mycotoxins: risks in plant, animal and human systems Ames, IA: Council for Agricultural Science and Technology; 2003.

[8] Yu JJ. Current Understanding on Aflatoxin Biosynthesis and Future Perspective in Reducing Aflatoxin Contamination. Toxins. 2012;4(11):1024–57. 10.3390/toxins4111024 23202305 PMC3509697

[9] van Egmond HP, Jonker MA, Abbas HK. Worldwide regulations on Aflatoxins. Aflatoxin and food safety. 2005;77–93.

[10] Lillehoj EB, Wall JH. Decontamination of aflatoxin-contaminated maize grain. In Proceedings of US Universities-CIMMYT Maize Aflatoxin Workshop, El Batan, Mexico.1987.

[11] Wright MS, Greene-McDowelle DM, Zeringue HJ, Bhatnagar D, Cleveland TE. Effects of volatile aldehydes from *Aspergillus*-resistant varieties of corn on *Aspergillus parasiticus* growth and aflatoxin biosynthesis. Toxicon. 2000;38(9):1215–23. 10736475 10.1016/s0041-0101(99)00221-4

[12] Cotty PJ. Influence of field application of an atoxigenic strain of *Aspergillus flavus* on the populations of *A*. *flavus* infecting cotton bolls and on the aflatoxin content of cottonseed. Phytopathology. 1994;84:1270–1277.

[13] Dorner JW. Biological control of aflatoxin contamination of crops. J Toxicol-Toxin Rev. 2004;23(2–3):425–50.

[14] Pitt JI, Hocking AD. Mycotoxins in Australia: biocontrol of aflatoxin in peanuts. Mycopathologia. 2006;162(3):233–43. 16944290 10.1007/s11046-006-0059-0

[15] Dorner JW. Management and prevention of mycotoxins in peanuts. Food Addit Contam. 2008;25(2):203–8. 10.1080/02652030701658357 18286410

[16] Bari R, Jones J. Role of plant hormones in plant defense responses. Plant Mol Biol. 2009;69(4):473–88. 10.1007/s11103-008-9435-0 19083153

[17] Birkenbihl RP, Somssich IE. Transcriptional plant responses critical for resistance towards necrotrophic pathogens. Front Plant Sci. 2011;2. 10.3389/fpls.2011.00076 PMC3355618 22639610

[18] St Clair DA. Quantitative Disease Resistance and Quantitative Resistance Loci in Breeding. Annu Rev Phytopathol. 2010;48:247–68. 10.1146/annurev-phyto-080508-081904 19400646

[19] Yu J, Bhatnagar D, Cleveland TE, Payne G, Nierman WC, Bennett JW. *Aspergillus flavus* genetics and genomics in solving mycotoxin contamination of food and feed. OMICs technologies: Tools for Food Science; 2012 pp 367–402.

[20] Guo BZ, Fedorova ND, Chen XP, Wan CH, Wang W, Nierman WC, et al Gene Expression Profiling and Identification of Resistance Genes to *Aspergillus flavus* Infection in Peanut through EST and Microarray Strategies. Toxins. 2011;3(7):737–5 10.3390/toxins3070737 22069737 PMC3202856

[21] Luo M, Brown RL, Chen ZY, Menkir A, Yu JJ, Bhatnagar D. Transcriptional Profiles Uncover *Aspergillus flavus*-Induced Resistance in Maize Kernels. Toxins. 2011;3(7):766–86. 10.3390/toxins3070766 22069739 PMC3202853

[22] Cleveland TE, Yu JJ, Bhatnagar D, Chen ZY, Brown RL, Chang PK, et al Progress in elucidating the molecular basis of the host plant—*Aspergillus flavus* interaction, a basis for devising strategies to reduce aflatoxin contamination in crops. J Toxicol-Toxin Rev. 2004;23(2–3):345–80.

[23] Grabherr MG, Haas BJ, Yassour M, Levin JZ, Thompson DA, Amit I, et al Full-length transcriptome assembly from RNA-Seq data without a reference genome. Nat Biotechnol. 2011;29(7):644–U130. 10.1038/nbt.1883 21572440 PMC3571712

[24] Altschul SF, Madden TL, Schaffer AA, Zhang JH, Zhang Z, Miller W, et al Gapped BLAST and PSI-BLAST: a new generation of protein database search programs. Nucleic Acids Res. 1997;25(17):3389–402. 9254694 10.1093/nar/25.17.3389 PMC146917

[25] Du Z, Zhou X, Ling Y, Zhang ZH, Su Z. agriGO: a GO analysis toolkit for the agricultural community. Nucleic Acids Res. 2010;38:W64–W70. 10.1093/nar/gkq310 20435677 PMC2896167

[26] Goffard N, Weiller G. PathExpress: a web-based tool to identify relevant pathways in gene expression data. Nucleic Acids Res. 2007;35:W176–W81. 17586825 10.1093/nar/gkm261 PMC1933187

[27] Wang KB, Wang ZW, Li FG, Ye WW, Wang JY, Song GL, et al The draft genome of a diploid cotton *Gossypium raimondii* . Nat Genet. 2012;44(10):1098–1103. 10.1038/ng.2371 22922876

[28] Li FG, Fan GY, Wang KB, Sun FM, Yuan YL, Song GL, et al Genome sequence of the cultivated cotton *Gossypium arboreum* . Nat Genet. 2014;46(6):567–72. 10.1038/ng.2987 24836287

[29] Trapnell C, Pachter L, Salzberg SL. TopHat: discovering splice junctions with RNA-Seq. Bioinformatics. 2009;25(9):1105–11. 10.1093/bioinformatics/btp120 19289445 PMC2672628

[30] Langmead B, Salzberg SL. Fast gapped-read alignment with Bowtie 2. Nat Methods. 2012;9(4):357–U54. 10.1038/nmeth.1923 22388286 PMC3322381

[31] Trapnell C, Williams BA, Pertea G, Mortazavi A, Kwan G, van Baren MJ, et al Transcript assembly and quantification by RNA-Seq reveals unannotated transcripts and isoform switching during cell differentiation. Nat Biotechnol. 2010;28(5):511–U174. 10.1038/nbt.1621 20436464 PMC3146043

[32] Trapnell C, Hendrickson DG, Sauvageau M, Goff L, Rinn JL, Pachter L. Differential analysis of gene regulation at transcript resolution with RNA-seq. Nat Biotechnol. 2013;31(1):46–53. 10.1038/nbt.2450 23222703 PMC3869392

[33] Drew DP, Dueholm B, Weitzel C, Zhang Y, Sensen CW, Simonsen HT. Transcriptome Analysis of *Thapsia laciniata* Rouy Provides Insights into Terpenoid Biosynthesis and Diversity in Apiaceae. Int J Mol Sci. 2013;14(5):9080–98. 10.3390/ijms14059080 23698765 PMC3676774

[34] Cardoso-Silva CB, Costa EA, Mancini MC, Balsalobre TWA, Canesin LEC, Pinto LR, et al De Novo Assembly and Transcriptome Analysis of Contrasting Sugarcane Varieties. Plos One. 2014;9(2). 10.1371/journal.pone.0088462 PMC3921171 24523899

[35] Tohidfar M, Mohammadi M, Ghareyazie B. *Agrobacterium*-mediated transformation of cotton (*Gossypium hirsutum*) using a heterologous bean chitinase gene. Plant Cell Tiss Org Cult. 2005;83(1):83–96.

[36] Wang SY, Ye XY, Chen J, Rao PF. A novel chitinase isolated from *Vicia faba* and its antifungal activity. Food Res Int. 2012;45(1):116–22.

[37] Collinge DB, Slusarenko AJ. Plant Gene-Expression in response to pathogens. Plant Mol Biol. 1987;9(4):389–410. 10.1007/BF00014913 24277091

[38] Prasad K, Bhatnagar-Mathur P, Waliyar F, Sharma KK. Overexpression of a chitinase gene in transgenic peanut confers enhanced resistance to major soil borne and foliar fungal pathogens. J Plant Biochem Biotechnol. 2013;22(2):222–33.

[39] Rohini VK, Rao KS. Transformation of peanut (*Arachis hypogaea* L.) with tobacco chitinase gene: variable response of transformants to leaf spot disease. Plant Sci. 2001;160(5):889–98. 11297785 10.1016/s0168-9452(00)00462-3

[40] Baisakh N, Datta K, Oliva N, Ona I, Rao GJN, Mew TW, et al Rapid development of homozygous transgenic rice using anther culture harboring rice chitinase gene for enhanced sheath blight resistance. Plant Biotechnol. 2001;18:101–8.

[41] Chen ZY, Brown RL, Russin JS, Lax AR. Cleveland TE. A corn trypsin inhibitor with antifungal activity inhibits *Aspergillus flavus* α-amylase. Phytopathology. 1999;89:902–907. 10.1094/PHYTO.1999.89.10.902 18944733

[42] Lawton MA, Lamb CJ. Transcriptional Activation of Plant Defense Genes by Fungal Elicitor, Wounding, and Infection. Mol Cell Biol. 1987;7(1):335–41. 3561393 10.1128/mcb.7.1.335-341.1987 PMC365073

[43] Chappell J, Hahlbrock K. Transcription of plant defense genes in response to UV-Light or fungal elicitor. Nature. 1984;311(5981):76–8.

[44] Ecker JR, Davis RW. Plant Defense Genes Are Regulated by Ethylene. P Natl Acad Sci USA. 1987;84(15):5202–6. 10.1073/pnas.84.15.5202 PMC298822 16593860

[45] HammondKosack KE, Jones JDG. Resistance gene-dependent plant defense responses. Plant Cell. 1996;8(10):1773–91. 8914325 10.1105/tpc.8.10.1773 PMC161314

[46] Cheong YH, Chang HS, Gupta R, Wang X, Zhu T, Luan S. Transcriptional profiling reveals novel interactions between wounding, pathogen, abiotic stress, and hormonal responses in Arabidopsis. Plant Physiol. 2002;129(2):661–77. 12068110 10.1104/pp.002857 PMC161692

[47] Beardmore J, Ride JP, Granger JW. Cellular Lignification as a Factor in the Hypersensitive Resistance of Wheat to Stem Rust. Physiol Plant Pathol. 1983;22(2):209.

[48] Sanchez-Vallet A, Lopez G, Ramos B, Delgado-Cerezo M, Riviere MP, Llorente F, et al Disruption of Abscisic Acid Signaling Constitutively Activates Arabidopsis Resistance to the Necrotrophic Fungus *Plectosphaerella cucumerina* . Plant Physiol. 2012;160(4):2109–24. 10.1104/pp.112.200154 23037505 PMC3510135

[49] Dolezal AL, Shu XM, OBrian GR, Nielsen DM, Woloshuk CP, Boston RS, et al *Aspergillus flavus* infection induces transcriptional and physical changes in developing maize kernels. Front Microbiol. 2014;5. 10.3389/fmicb.2014.00384 PMC4117183 25132833

[50] Garcia-Brugger A, Lamotte O, Vandelle E, Bourque S, Lecourieux D, Poinssot B, et al Early signaling events induced by elicitors of plant Defenses. Mol Plant Microbe In. 2006;19(7):711–24. 10.1094/MPMI-19-0711 16838784

[51] Tena G, Boudsocq M, Sheen J. Protein kinase signaling networks in plant innate immunity. Curr Opin Plant Biol. 2011;14(5):519–29. 10.1016/j.pbi.2011.05.006 21704551 PMC3191242

[52] Dodds PN, Rathjen JP. Plant immunity: towards an integrated view of plant-pathogen interactions. Nat Rev Genet. 2010;11(8):539–48. 10.1038/nrg2812 20585331

[53] Asano T, Hayashi N, Kikuchi S, Ohsugi R. CDPK-mediated abiotic stress signaling. Plant Signaling & Behavior; 2012;7:817–21. 22751324 10.4161/psb.20351 PMC3583972

[54] Yoshioka H, Mase K, Yoshioka M, Kobayashi M, Asai S. Regulatory mechanisms of nitric oxide and reactive oxygen species generation and their role in plant immunity. Nitric Oxide-Biol Ch. 2011;25(2):216–21. 10.1016/j.niox.2010.12.008 21195205

[55] Wang P, Du Y, Li Y, Ren D, Song CP. Hydrogen peroxide–mediated activation of MAP kinase 6 modulates nitric oxide biosynthesis and signal transduction in *Arabidopsis* . The Plant Cell Online. 2010;22:2981–98. 10.1105/tpc.109.072959 PMC2965546 20870959

[56] Asai S, Ohta K, Yoshioka H. MAPK signaling regulates nitric oxide and NADPH oxidase-dependent oxidative bursts in *Nicotiana benthamiana* . Plant Cell. 2008;20(5):1390–406. 10.1105/tpc.107.055855 18515503 PMC2438462

[57] Torres MA. ROS in biotic interactions. Physiol Plant. 2010;138(4):414–29. 10.1111/j.1399-3054.2009.01326.x 20002601

[58] Ghanashyam C, Jain M. Role of auxin-responsive genes in biotic stress responses. Plant Signal Behav. 2009;4:846–48. 19847104 10.4161/psb.4.9.9376 PMC2802801

[59] Domingo C, Andres F, Tharreau D, Iglesias DJ, Talon M. Constitutive Expression of OsGH3.1 Reduces Auxin Content and Enhances Defense Response and Resistance to a Fungal Pathogen in Rice. Mol Plant Microbe In. 2009;22(2):201–10. 10.1094/MPMI-22-2-0201 19132872

[60] Kant S, Bi YM, Zhu T, Rothstein SJ. SAUR39, a Small Auxin-Up RNA Gene, Acts as a Negative Regulator of Auxin Synthesis and Transport in Rice. Plant Physiol. 2009;151(2):691–701. 10.1104/pp.109.143875 19700562 PMC2754634

[61] Siemens J, Keller I, Sarx J, Kunz S, Schuller A, Nagel W, et al Transcriptome analysis of Arabidopsis clubroots indicate a key role for cytokinins in disease development. Mol Plant Microbe In. 2006;19(5):480–94. 10.1094/MPMI-19-0480 16673935

[62] Guo RY, Yu FF, Gao Z, An HL, Cao XC, Guo XQ. GhWRKY3, a novel cotton (*Gossypium hirsutum* L.) WRKY gene, is involved in diverse stress responses. Mol Biol Rep. 2011;38(1):49–58. 10.1007/s11033-010-0076-4 20238169

[63] Du L, Chen Z. Identification of genes encoding receptor‐like protein kinases as possible targets of pathogen‐and salicylic acid‐induced WRKY DNA‐binding proteins in *Arabidopsis* . Plant J. 2000;24:837–47. 11135117 10.1046/j.1365-313x.2000.00923.x

[64] Lorenzo O, Piqueras R, Sanchez-Serrano JJ, Solano R. Ethylene Response Factor1 Integrates Signals from Ethylene and Jasmonate Pathways in Plant Defense. Plant Cell. 2003;15(1):165–78. 12509529 10.1105/tpc.007468 PMC143489

[65] Singh KB, Foley RC, Onate-Sanchez L. Transcription factors in plant defense and stress responses. Curr Opin Plant Biol. 2002;5(5):430–6. 12183182 10.1016/s1369-5266(02)00289-3

[66] Bolwell GP, Wojtaszek P. Mechanisms for the generation of reactive oxygen species in plant defense—a broad perspective. Physiol Mol Plant P. 1997;51(6):347–66.

[67] Guan X, Zhao HQ, Xu Y, Wang YJ. Transient expression of glyoxal oxidase from the Chinese wild grape *Vitis pseudoreticulata* can suppress powdery mildew in a susceptible genotype. Protoplasma. 2011;248(2):415–23. 10.1007/s00709-010-0162-4 20512385

[68] Coumans JVF, Polijak A, Raftery MJ, Backhouse D, Pereg-Gerk L. Analysis of cotton (*Gossypium hirsutum*) root proteomes during a compatible interaction with the black root rot fungus *Thielaviopsis basicola* . Proteomics. 2009;9(2):335–49. 10.1002/pmic.200800251 19105169

[69] Pritsch C, Muehlbauer GJ, Bushnell WR, Somers DA, Vance CP. Fungal development and induction of defense response genes during early infection of wheat spikes by *Fusarium graminearum* . Mol Plant Microbe In. 2000;13(2):159–69. 10.1094/MPMI.2000.13.2.159 10659706

[70] Treutter D. Significance of flavonoids in plant resistance and enhancement of their biosynthesis. Plant Biology. 2005;7(6):581–91. 16388461 10.1055/s-2005-873009

[71] Todd AT, Liu EW, Polvi SL, Pammett RT, Page JE. A functional genomics screen identifies diverse transcription factors that regulate alkaloid biosynthesis in *Nicotiana benthamiana* . Plant J. 2010;62(4):589–600. 10.1111/j.1365-313X.2010.04186.x 20202168

[72] Savchenko T, Walley JW, Chehab EW, Xiao YM, Kaspi R, Pye MF, et al Arachidonic Acid: An Evolutionarily Conserved Signaling Molecule Modulates Plant Stress Signaling Networks. Plant Cell. 2010;22(10):3193–205. 10.1105/tpc.110.073858 20935246 PMC2990140

[73] Wasternack C. Action of jasmonates in plant stress responses and development applied aspects. Biotechnol Adv. 2014;32(1):31–9. 10.1016/j.biotechadv.2013.09.009 24095665

[74] Robert-Seilaniantz A, Grant M, Jones JDG. Hormone Crosstalk in Plant Disease and Defense: More than just JASMONATE-SALICYLATE antagonism. Annu. Rev. Phytopathol., 2011;49:317–43. 10.1146/annurev-phyto-073009-114447 21663438

[75] Rojas CM, Senthil-Kumar M, Tzin V, Mysore KS. Regulation of primary plant metabolism during plant-pathogen interactions and its contribution to plant defense. Front Plant Sci. 2014;5. 10.3389/fpls.2014.00017 PMC3919437 24575102

