# Supplementary figures and images for: Genome-Wide Transcriptome Analysis of Cotton (Gossypium hirsutum L.) Identifies Candidate Gene Signatures in Response to Aflatoxin Producing Fungus Aspergillus flavus
Source: PLoS One. 2015 Sep 14;10(9):e0138025. doi: 10.1371/journal.pone.0138025 (PMC4569580; doi:10.1371/journal.pone.0138025)

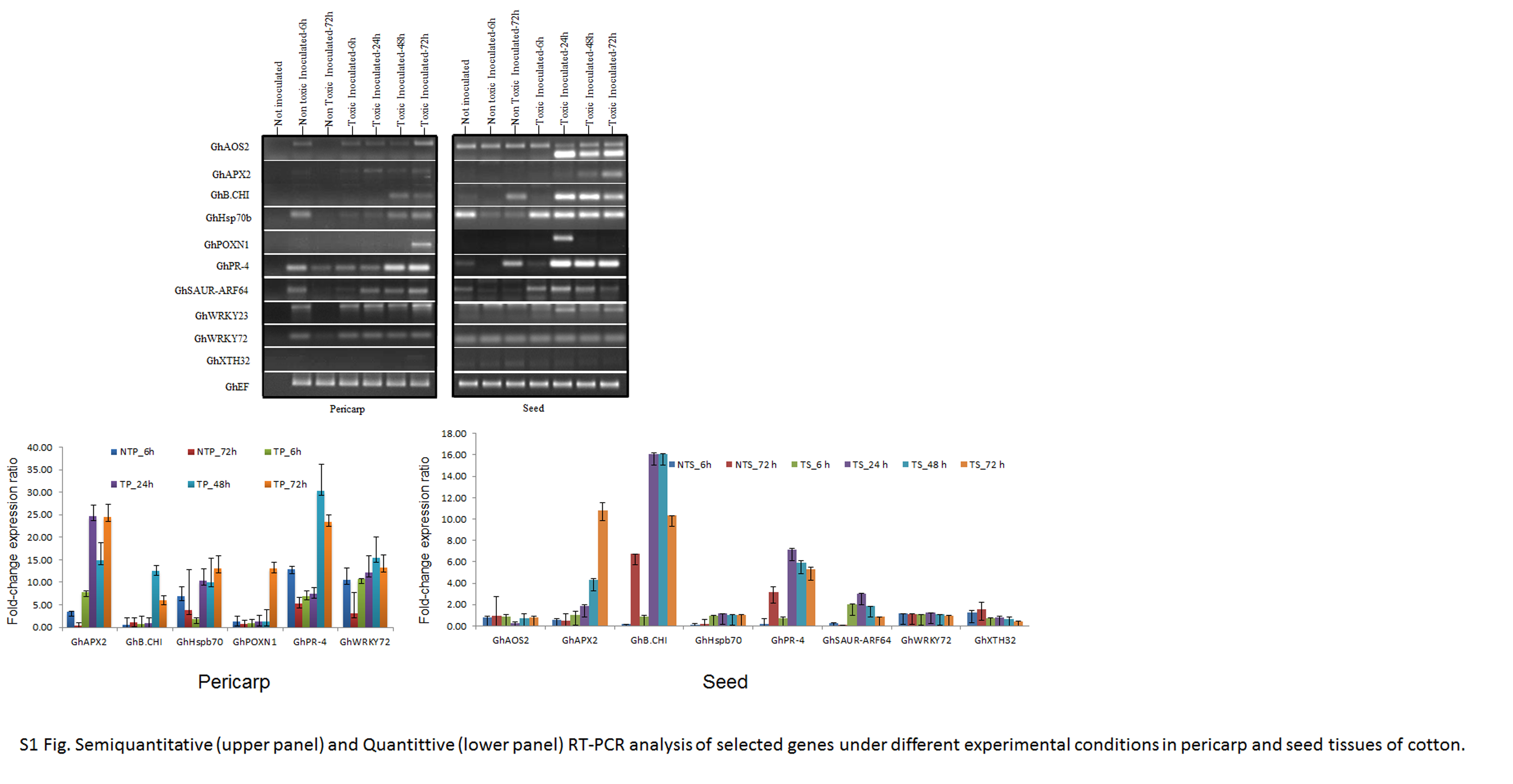

Supplement: S1 Fig — Gene expression was normalized using cotton elongation factor [4] as the internal reference against the expression in non-inoculated control, which was set to 1. (TIF) [file pone.0138025.s001.tif]
